# Tailoring the pore chemistry in porous aromatic frameworks for selective separation of acetylene from ethylene†

**DOI:** 10.1039/d2sc03944c

**Published:** 2022-08-20

**Authors:** Shuang Zhou, Zhaoli Liu, Panpan Zhang, Huazhen Rong, Tingting Ma, Fengchao Cui, Dongtao Liu, Xiaoqin Zou, Guangshan Zhu

**Affiliations:** Department of Chemistry, Northeast Normal University Changchun Jilin 130024 P. R. China liudt737@nenu.edu.cn zouxq100@nenu.edu.cn zhugs100@nenu.edu.cn

## Abstract

The separation of acetylene from ethylene is a crucial process in the petrochemical industry, because even traces of acetylene impurities can poison the catalysts of ethylene polymerization. Herein, we synthesize a new family of 3D porous aromatic frameworks (PAFs), non-functionalized PAF-28, carbene-functionalized PAF-28 (cPAF-28) and imidazolium-functionalized PAF-28 (iPAF-28), *via* Sonogashira coupling reactions. These PAFs show high porosity and good thermal stability. Both cPAF-28 and iPAF-28 are proved to be good candidates for C_2_H_2_ adsorption, demonstrated by C_2_H_2_/C_2_H_4_ selectivity of 12.2 and 15.4, and C_2_H_2_ capacity of 48 cm^3^ g^−1^ and 57 cm^3^ g^−1^, which are significantly higher than those of non-functionalized PAF-28 (1.8, 37 cm^3^ g^−1^). Furthermore, the cPAF-28 and iPAF-28 display good breakthrough performance and remarkable recyclability for the separation of the C_2_H_2_/C_2_H_4_ gas mixture. In addition, the C_2_H_2_/C_2_H_4_ adsorption sites are revealed by DFT calculations. This work sheds a new light on gas molecular recognition by tailoring the pore chemistry of PAFs.

## Abstract 2

A family of PAFs with 3D topology was developed through tailoring the pore structures. By anchoring carbene or imidazolium groups in PAF-28, the selectivity for separating acetylene from ethylene was greatly boosted from 1.8 to 15.4.

## Graphical abstract

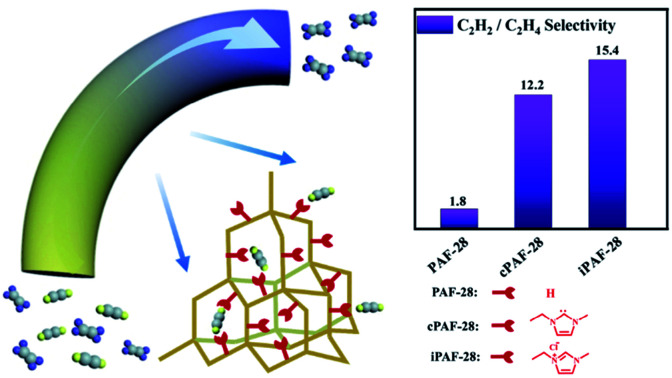

## Introduction

Ethylene (C_2_H_4_), the most produced organic compound in the world with an annual production capacity of 180 million tons in 2019, is an essential raw material for the manufacture of many polymer products.^1^ Ethylene is mainly produced from steam cracking of petroleum-based hydrocarbons in the petrochemical industry. A small amount of acetylene (C_2_H_2_) is unavoidably generated as a byproduct of this process and poisons the Ziegler–Natta catalysts of ethylene polymerization by forming stable metal acetylides.^2^ On the other hand, acetylene is also one of the most important C_2_ hydrocarbons and is used to produce many chemicals (*i.e.*, vinyl chloride and polyacetylene).^3^ Therefore, separating C_2_H_2_ from C_2_H_4_ is of great significance. However, the C_2_H_2_/C_2_H_4_ separation is a long-standing challenge because they have similar kinetic diameters (C_2_H_2_: 3.3 Å *vs.* C_2_H_4_: 4.1 Å) and similar physicochemical properties.^4^ Current strategies for the separation of C_2_H_2_ from C_2_H_4_ in industrial processes are mainly cryogenic distillation, catalytic hydrogenation of C_2_H_2_ to C_2_H_4_, and solvent extraction.^5^ These processes are either energy-/cost-intensive or environmentally unfriendly. Therefore, developing more energy-efficient technologies for carrying out the separation of C_2_H_2_ from C_2_H_4_ is highly desirable.

Physisorption-based separation using porous materials has attracted tremendous attention for gaseous hydrocarbon separation because of its efficient and eco-friendly process.^6^ As a class of important porous materials, metal–organic frameworks (MOFs) have been well developed for C_2_H_2_/C_2_H_4_ separation, and high selectivity has been achieved owing to their well-defined adsorptive sites and fine-tuned pore sizes.^7^ Recently, a class of ionic ultramicroporous polymers was reported through free-radical polymerization of branched ionic monomers, affording highly selective recognition for the C_2_H_2_/C_2_H_4_ mixture but with relatively low gas adsorption capacity.^8^

Porous aromatic frameworks (PAFs), an emerging class of porous materials, are built from organic aromatic monomers and linked by strong covalent bonds to form periodically open and extended structures.^9^ PAFs are known for their rigid structures, high surface areas, exceptional thermal stabilities, and versatile functionalities, and have widespread applications, such as in gaseous hydrocarbon adsorption and separation.^10^ Crystalline PAF-110 (ref. 11) and PAF-120,^12^ generated from oxygen-containing aromatic monomers, were explored for C_2_H_2_/C_2_H_4_ separation with moderate selectivity (C_2_H_2_/C_2_H_4_: 3.9–4.1) due to their weak adsorption sites. Very recently, an anion-substitution strategy of ionic PAF-1 (iPAF-1) was introduced to improve the selectivity for C_2_H_2_/C_2_H_4_ separation.^13^ A high C_2_H_2_/C_2_H_4_ selectivity of 9.99 was achieved for iPAF-1-OH. Despite these achievements, exploring new functional PAFs with better C_2_H_2_/C_2_H_4_ separation performance is still an open research area, and in particular, the influences of different functional groups in PAFs on the C_2_H_2_/C_2_H_4_ adsorption and separation are still little known.

In the present work, we report a family of new PAFs with 3D topology obtained by task-specific design of different substituent groups. Firstly, we designed a parent material affording non-functionalized PAF-28 *via* the Sonogashira coupling reaction of tetrakis(4-ethynylphenyl)methane with 1,4-dibromobenzene. Subsequently, cPAF-28 with carbene functional groups and iPAF-28 with imidazolium functional groups were synthesized by introducing two wall-mounted basic substituents in PAF-28, because the carbenes and imidazoles are important organic bases, which are promising adsorbents for acidic acetylene. The obtained PAFs showed high porosity and good thermal stability. The effects of different pore chemistries in these PAFs on the selective adsorption of acetylene were investigated. PAF-28 had good adsorption for both C_2_H_2_ and C_2_H_4_ due to its large pore size distribution but with a low C_2_H_2_/C_2_H_4_ selectivity of 1.8. In contrast, both cPAF-28 and iPAF-28 were proved to be good candidates for C_2_H_2_ adsorption and they exhibited excellent C_2_H_2_/C_2_H_4_ selectivity and high C_2_H_2_ capacity owing to their stronger adsorption sites and smaller pore sizes. The cPAF-28 and iPAF-28 also displayed good breakthrough performance for separating the C_2_H_2_/C_2_H_4_ gas mixture with high recyclability. Additionally, the C_2_H_2_/C_2_H_4_ adsorption sites in these PAFs were elucidated by DFT simulations.

## Results and discussion

**Fig. 1:**
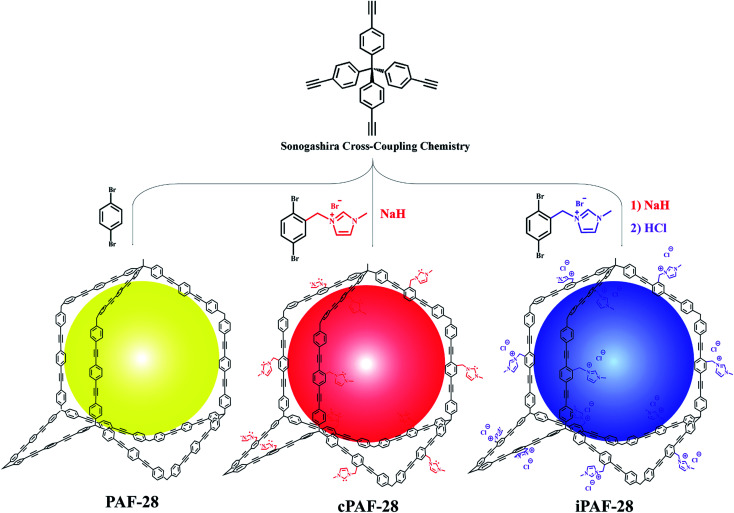
Schematic representation of the synthesis of PAF-28, cPAF-28 and iPAF-28.

**Fig. 2:**
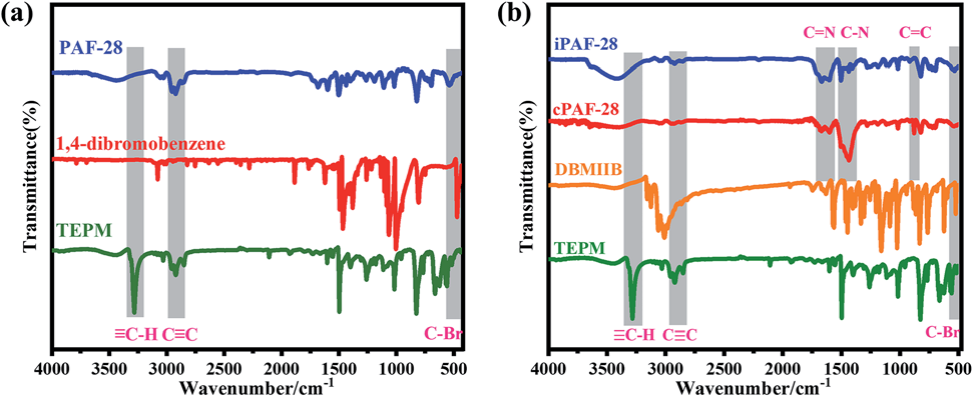
FTIR spectra of PAF-28 and its monomers (a), and cPAF-28 and iPAF-28 and their monomers (b).

Three-dimensional PAFs have shown great advantages in gas adsorption and separation owing to their interconnected pore structures and high stabilities.^10*a*,13^ We adopted the tetrahedral building block linked with modifiable linear ones to design 3D functional PAFs (PAF-28, cPAF-28 and iPAF-28) (Fig. 1). PAF-28 was built from tetrakis(4-ethynylphenyl)methane and 1,4-dibromobenzene. As shown in Fig. 2a, the infrared (IR) bands associated with C–Br vibrations at 473 cm^−1^ in 1,4-dibromobenzene and ≡C–H vibrations at 3300 cm^−1^ in tetrakis(4-ethynylphenyl)methane were no longer present in PAF-28, primarily indicating that the cross-coupling reactions for PAF-28 have successfully proceeded and completed.

N-Heterocyclic carbenes (NHCs) are stable heterocyclic species^14^ which contain a carbene carbon and two or more nitrogen atoms within the ring structure. As a Lewis base, NHC has been widely used in catalytic polymerization and organic synthesis.^15^ On the other hand, NHCs could be anchored in nanoporous materials and as promising adsorbents for Lewis acidic gases, such as acetylene, owing to their moderate alkalinity. So far, however, NHC functionalized porous materials have not yet been reported. In this work, tetrakis(4-ethynylphenyl)methane first reacted with 3-(2,5-dibromobenzyl)-1-methyl-1*H*-imidazol-3-ium bromide to form an intermediate which was subsequently treated with NaH to produce cPAF-28 with carbene groups. Interestingly, after reacting with HCl, cPAF-28 can be almost completely converted into iPAF-28 with imidazolium groups. The chemical structures of cPAF-28 and iPAF-28 were also characterized by IR spectra (Fig. 2b). The band at 1458 cm^−1^ for the C–N stretching vibration almost disappeared in iPAF-28, suggesting that C–N in cPAF-28 is transformed into C=N in iPAF-28.

**Fig. 3:**
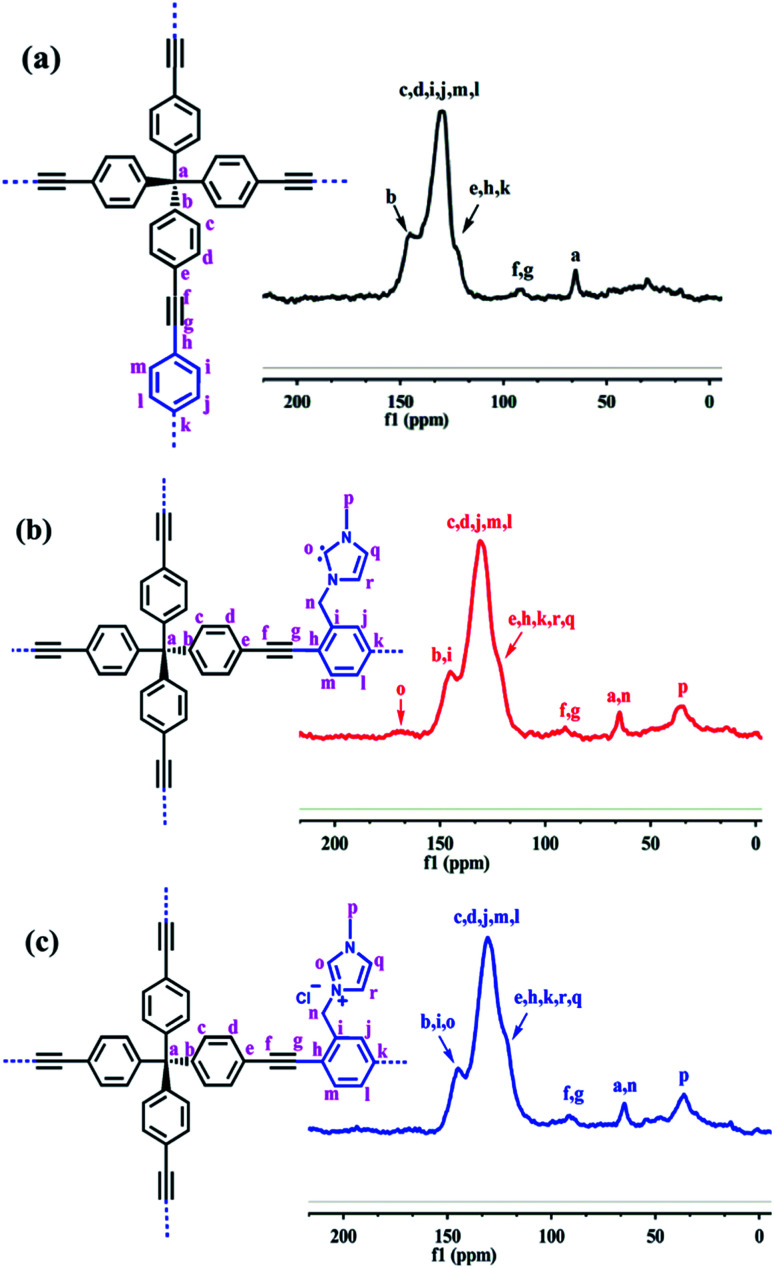
Solid-state ^13^C NMR spectra of (a) PAF-28, (b) cPAF-28 and (c) iPAF-28.

To further reveal the structure of these PAFs, the solid-state ^13^C NMR spectra for PAF-28, cPAF-28 and iPAF-28 were recorded (Fig. 3). In PAF-28, the intense signals at approximately *δ* = 145, 130 and 123 ppm can be assigned to the carbon atoms in the benzene rings, while the relatively weak signal at *δ* = 90 ppm is attributed to the alkynyl carbon (Fig. 3a). The signal at *δ* = 65 ppm can be assigned to the quaternary carbon atom that is connected to four phenyl groups. In addition to the above signals observed in PAF-28, the NMR spectrum of cPAF-28 contains two new signals at *δ* = 166 and 36 ppm (Fig. 3b). The signal at *δ* = 166 ppm can be assigned to the carbene carbon, comparable to the chemical shifts of other carbene carbons reported in the literature.^16^ The signal at *δ* = 36 ppm is assigned to the methyl carbon. The ^13^C NMR spectrum of iPAF-28 resembles that of cPAF-28 except for the disappearance of the carbene carbon signal at 166 ppm (Fig. 3c). The elemental compositions of PAF-28, cPAF-28 and iPAF-28 measured experimentally are close to the calculated values (Table S1†). Scanning electron microscopy (SEM) and transmission electron microscopy (TEM) images show that all three PAFs are composed of spherical particles 200 nm in diameter (Fig. S5†). Thermogravimetric analysis shows that the synthesized PAFs exhibit good thermal stability up to 300 °C (Fig. S7†). As shown in Fig. S10,† no distinct diffraction peaks were detected except for a broad peak for all three PAFs, indicating that the synthesized PAFs are amorphous structures.

**Fig. 4:**
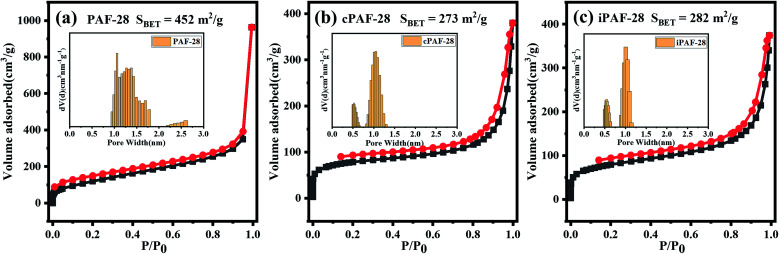
N_2_ adsorption–desorption isotherms measured at 77 K for PAF-28 (a), cPAF-28 (b) and iPAF-28 (c). Black and red symbols in the isotherms represent the adsorption and desorption branches, respectively. The insets are the corresponding pore size distributions.

The pore structures of PAF-28, cPAF-28 and iPAF-28 were investigated by physical nitrogen sorption. The N_2_ adsorption and desorption isotherms of the three PAFs at 77 K are shown in Fig. 4. The uptakes in all isotherms rise rapidly to about 70 cm^3^ g^−1^ at low relative pressures of *P*/*P*_0_ (≤0.003) and then increase slowly at medium *P*/*P*_0_ (0.003–0.85), a quasi-type I adsorption curve, indicating that the three PAFs have microporous structures. The increase of N_2_ uptake at *P*/*P*_0_ ≥ 0.85 is due to gas condensation in large pores between small particles. Calculated from the isotherms, the Brunauer–Emmett–Teller surface areas (*S*_BET_) are 452 m^2^ g^−1^, 273 m^2^ g^−1^ and 282 m^2^ g^−1^ for PAF-28, cPAF-28 and iPAF-28, respectively. The pore size distributions were calculated using the quenched-solid density functional theory (QSDFT) method (insets in Fig. 4). PAF-28 has 1.0–1.5 nm pores and even some large pores above 2.0 nm. The pores of the other two PAFs (cPAF-28 and iPAF-28) are mostly distributed at 0.6 nm and 1.0 nm. The pore narrowness is presumably because the substituents in PAFs occupy some pore space. To the best of our knowledge, cPAF-28 is the first example of carbene-functionalized microporous materials. The microporous structure and substituent-tailored environment inspire us to explore the function of PAFs for hydrocarbon adsorption and separation.

**Fig. 5:**
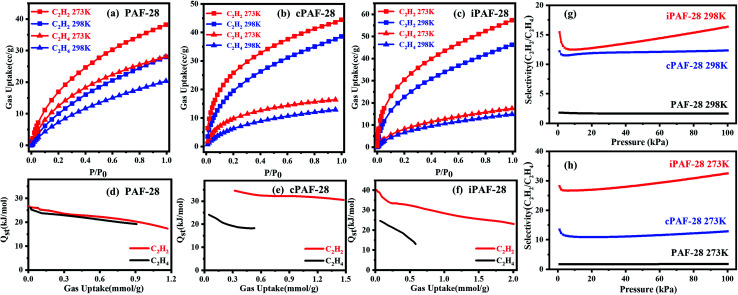
C_2_H_2_/C_2_H_4_ adsorption isotherms at 273 K and 298 K for PAF-28 (a), cPAF-28 (b) and iPAF-28 (c). *P*_0_ is 100 kPa. Isosteric heats of adsorption (*Q*_st_) of C_2_H_2_ and C_2_H_4_ as a function of gas uptake for PAF-28 (d), cPAF-28 (e) and iPAF-28 (f). The selectivity of C_2_H_2_/C_2_H_4_ predicted by IAST for PAF-28, cPAF-28 and iPAF-28 at 298 K (g) and 273 K (h).

The adsorptions of PAF-28, cPAF-28 and iPAF-28 for ethylene and acetylene were studied by single-component gas adsorption experiments at 273 K and 298 K. The three PAFs exhibit higher uptake for C_2_H_2_ than C_2_H_4_, especially for cPAF-28 and iPAF-28 (Fig. 5a–c). Specifically, the C_2_H_2_ uptakes are 48 cm^3^ g^−1^ and 57 cm^3^ g^−1^ for cPAF-28 and iPAF-28, respectively at 273 K and 100 kPa, which are much higher than that for PAF-28 (37 cm^3^ g^−1^). The high gas uptake is attributed to the massive pores in which C_2_H_2_ or C_2_H_4_ molecules are closely packed. The preferential adsorption for C_2_H_2_ can be rationalized by the acid–base interaction: N-heterocyclic compounds are commonly considered as Lewis bases and C_2_H_2_ gas is a typical Lewis acid. As two types of N-heterocyclic compounds, both carbene and imidazolium moieties show moderate basicity. For example, 1,3-diphenylimidazol-2-ylidene and 1,3-diphenylimidazolium chloride have p*K*_a_ values of 16.1 (ref. 17) and 18.3 (ref. 18) in dimethyl sulfoxide, which are significantly higher than that of benzene (p*K*_a_ = 9.3). PAF-28 consists of phenyl rings, while cPAF-28 and iPAF-28 contain imidazole carbenes and imidazole salts. Thus, PAF-28, cPAF-28 and iPAF-28 in principle have the sequential basicity of PAF-28 < cPAF-28 ∼ iPAF-28. The frameworks of cPAF-28 and iPAF-28 bearing carbene and imidazolium substituents can form stronger acid–base pairs with C_2_H_2_ than PAF-28. To prove this hypothesis, the isosteric heats of adsorption (*Q*_st_) of C_2_H_2_ and C_2_H_4_ for PAF-28 (Fig. 5d), cPAF-28 (Fig. 5e) and iPAF-28 (Fig. 5f) were quantitatively obtained from the adsorption isotherms at 273 K and 298 K. *Q*_st_ values of C_2_H_2_ are 35.0 kJ mol^−1^ (cPAF-28) and 40.0 kJ mol^−1^ (iPAF-28) at low uptakes, larger than that of PAF-28 (26.7 kJ mol^−1^), indicating that basic substituents can enhance the host–guest interactions between C_2_H_2_ and PAFs. It should be noted that the C_2_H_2_*Q*_st_ for cPAF-28 and iPAF-28 are comparable to those of some previously reported MOFs, such as FJI-H8 (32.0 kJ mol^−1^),^19^ SIFSIX-2-Cu-I (41.9 kJ mol^−1^),^20^ NOTT-300 (32 kJ mol^−1^),^21^ SNNU-45 (40 kJ mol^−1^),^22^ and HKUST-1 (30.4 kJ mol^−1^).^23^ The thermodynamic selectivity for C_2_H_2_ over C_2_H_4_ was quantified by ideal adsorbed solution theory (IAST) at 298 K and 273 K (Fig. 5g and h). The predicted selectivity for C_2_H_2_ over C_2_H_4_ increases from 1.8 (PAF-28) to 12.2 (cPAF-28) and 15.4 (iPAF-28) at 100 kPa and 298 K. The selectivity increase can be attributed to the fact that basic substituents amplify the interaction of the PAF with C_2_H_2_ rather than C_2_H_4_, consistent with the *Q*_st_ results (C_2_H_2_*Q*_st_ from 26.7 kJ mol^−1^ (PAF-28) to 40.0 kJ mol^−1^ (iPAF-28), C_2_H_4_*Q*_st_ from 25 kJ mol^−1^ (PAF-28) to 26.5 kJ mol^−1^ (iPAF-28)). Compared with cPAF-28, the slightly higher selectivity observed for iPAF-28 is probably because the imidazolium substituent and chloride anion of iPAF-28 may simultaneously interact with C_2_H_2_. It can be concluded from the above results that the basic substituents introduced onto PAF backbones effectively tailor the pore chemistry to improve the selectivity of C_2_H_2_ adsorption.

**Fig. 6:**
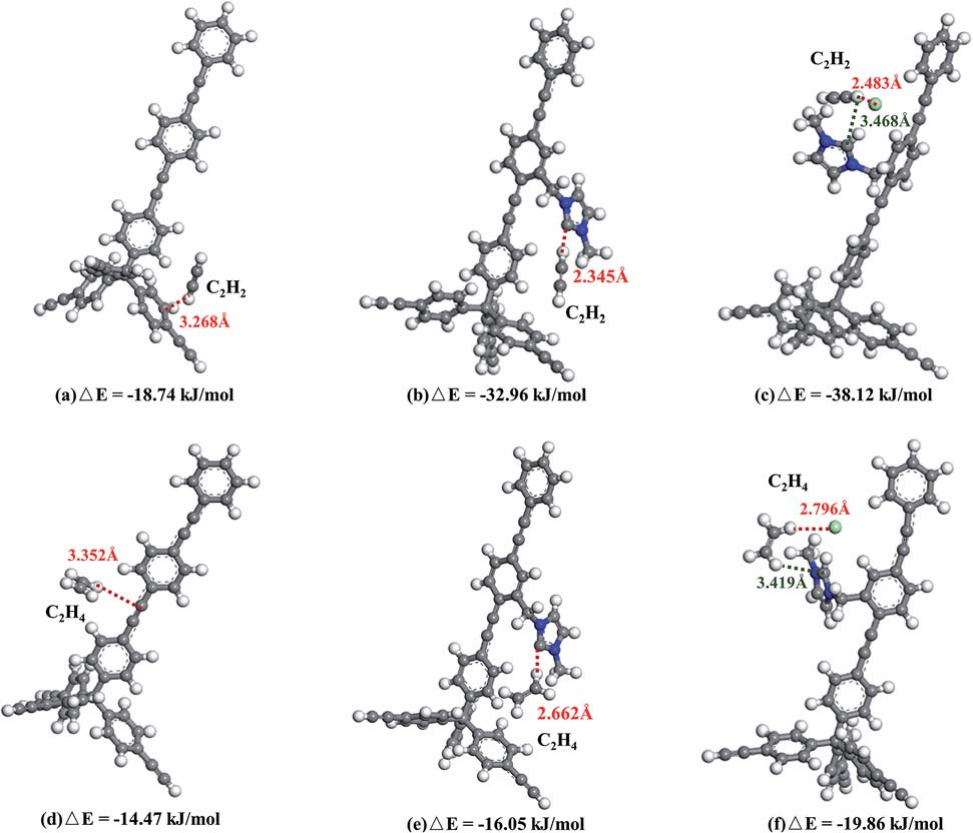
Preferred binding sites and calculated binding energies of PAF-28 (a and d), cPAF-28 (b and e) and iPAF-28 (c and f) for C_2_H_2_ and C_2_H_4_.

DFT calculations were performed to provide information on adsorption sites and to unveil the adsorption sites (Fig. 6). Both C_2_H_2_ and C_2_H_4_ molecules electrostatically bind with the PAF frameworks. However, the binding sites in PAF-28, cPAF-28 and iPAF-28 are different and binding energies with C_2_H_2_ and C_2_H_4_ vary as well. After structural optimization, the C_2_H_2_ molecule resides around the phenyl ring in PAF-28, giving rise to a small binding energy (Δ*E*) of −18.74 kJ mol^−1^. The similar values of C_2_H_2_ and C_2_H_4_ binding energy with PAF-28 explain the low C_2_H_2_/C_2_H_4_ selectivity measured by gas adsorption. In cPAF-28, the carbene carbon becomes the preferential binding site which strongly binds the C_2_H_2_ molecule with a distance of 2.345 Å and an energy of −32.96 kJ mol^−1^. Moreover, carbene is able to recognize C_2_H_2_ over C_2_H_4_, shown by the enlarged difference of cPAF28-C_2_H_2_ and cPAF28-C_2_H_4_ binding energies. This finding agrees well with the significant increase of C_2_H_2_/C_2_H_4_ adsorption selectivity. As can be seen, after the carbene reacts with HCl, iPAF-28 possesses two optimal binding sites of imidazolium ring and chloride ion, which synergically bind with hydrogen in C_2_H_2_. This synergic effect gives a more negative C_2_H_2_ Δ*E* value of −38.12 kJ mol^−1^, resulting in more selective adsorption for C_2_H_2_ over C_2_H_4_ by iPAF-28.

**Fig. 7:**
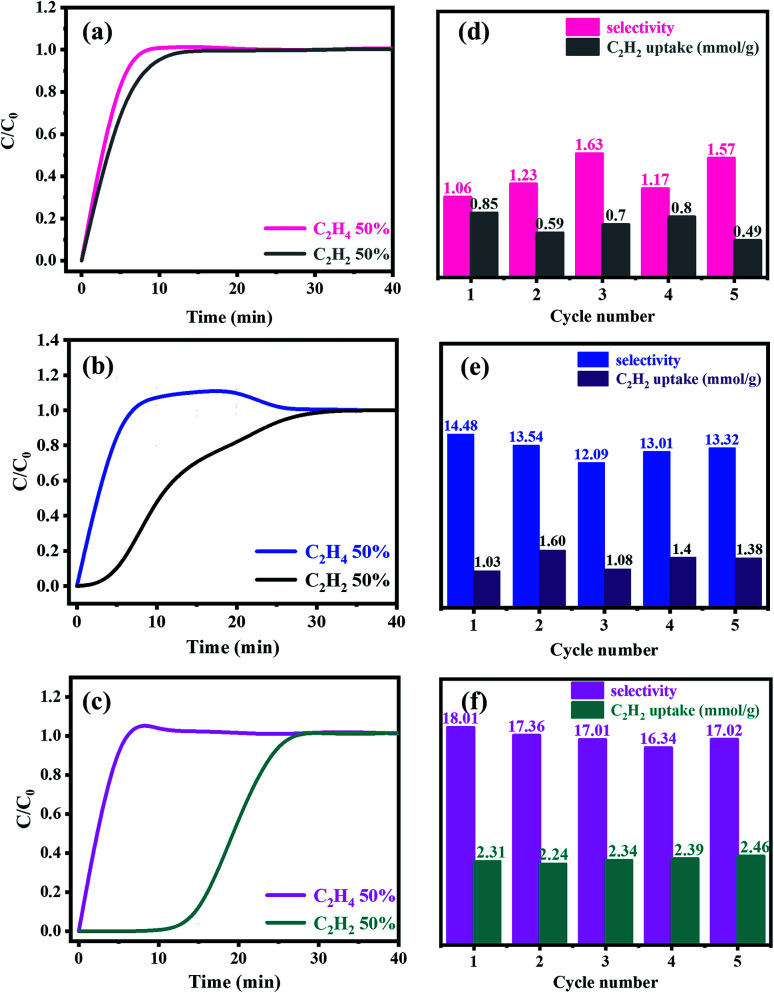
Column breakthrough curves of the C_2_H_2_/C_2_H_4_ gas mixture (50/50, v/v) with PAF-28 (a), cPAF-28 (b) and iPAF-28 (c) at 298 K and 1 atm. Recyclability of PAF-28 (d), cPAF-28 (e) and iPAF-28 (f) in terms of C_2_H_2_/C_2_H_4_ selectivity and C_2_H_2_ capacity.

Further, the separation potential of C_2_H_2_/C_2_H_4_ was evaluated by dynamic sorption of the gas mixture. Column breakthrough measurements of C_2_H_2_ and C_2_H_4_ were carried out on PAF materials. Prior to the measurement, PAF-28, cPAF-28 and iPAF-28 were pressed and ground into ∼100 μm particles in order to avoid any gas resistance in the column. These particles (∼1.0 g) were subsequently packed in the column, and the C_2_H_2_/C_2_H_4_ gas mixture (50/50, v/v) was passed through the PAF-packed column at a rate of 3 ml min^−1^ at 298 K and atmospheric pressure (1 bar). The breakthrough curves are compiled in Fig. 7. For PAF-28, C_2_H_4_ and C_2_H_2_ almost elute through the bed simultaneously (Fig. 7a), leading to a selectivity of 1.06 for C_2_H_2_/C_2_H_4_ (Fig. 7d). This observation is consistent with the similar binding energies of C_2_H_2_ and C_2_H_4_ with PAF-28 (Fig. 6a and d). For cPAF-28, C_2_H_4_ elutes quickly through the bed; in contrast, C_2_H_2_ is retained in the column for about 5 minutes (Fig. 7b). Integration of the curve area gives a C_2_H_2_/C_2_H_4_ selectivity of 14.5 for cPAF-28. Selective separation of C_2_H_2_ from C_2_H_4_ is ascribed to the binding preference of the C_2_H_2_ molecule with the cPAF-28 framework. iPAF-28 exhibits longer retention toward C_2_H_2_ (>10 min) than cPAF-28 (∼5 min) (Fig. 7c), indicating that C_2_H_2_ interacts more strongly with iPAF-28, in agreement with the more negative binding energy (Fig. 6c and b). Consequently, iPAF-28 has a higher C_2_H_2_/C_2_H_4_ selectivity with a value of 18.01 (Fig. 7f). The dynamic adsorption capacity of iPAF-28 for C_2_H_2_ is estimated to be 2.3 mmol g^−1^, which is similar to that measured under static conditions (2.1 mmol g^−1^ at 298 K), reflecting that this gas separation is based on the adsorption mechanism. All three PAFs are able to stably separate C_2_H_2_ from C_2_H_4_, shown by the invariable selectivity and capacity during five-cycle tests (Fig. 7d–f). From this aspect, the functionalized PAFs are promising alternatives for C_2_H_2_/C_2_H_4_ separation with high selectivity and excellent stability.

## Conclusions

In summary, we have developed a new family of functionalized PAFs with 3D structures. Anchoring carbene or imidazolium groups in PAF-28 afforded cPAF-28 and iPAF-28 with smaller pore size, higher basicity and higher thermal stability. Moreover, the selectivity of C_2_H_2_/C_2_H_4_ was greatly boosted from 1.8 (PAF-28) to 12.2 (cPAF-28) and to 15.4 (iPAF-28), owing to the preferential adsorption of C_2_H_2_. DFT simulations demonstrated that the carbene or imidazolium bound C_2_H_2_ much stronger than C_2_H_4_. Furthermore, the column breakthrough experiments and cycling tests confirmed the superior efficiency and outstanding stability of cPAF-28 and iPAF-28 for C_2_H_2_/C_2_H_4_ gas separation, which are critical for industrial application. This work provides an effective and feasible route for C_2_H_2_/C_2_H_4_ adsorption and separation by tailoring the pore chemistry of PAFs, and may pave the way for future development of separation strategies for other target molecules.

## Data availability

All relevant data are available from the corresponding authors upon reasonable request.

## Author contributions

D. L. and G. Z. initiated and designed this work. X. Z. and S. Z. devised the gas separation and wrote this paper. S. Z. and P. Z. conducted the synthesis and characterization of the materials. F. C. and Z. L. conducted the computational work. T. M. and H. R. helped with the characterization of the materials. X. Z. and D. L. revised this paper.

## Conflicts of interest

There are no conflicts to declare.

## Supplementary Material

SC-013-D2SC03944C-s001

## References

[cit1] Li L., Lin R., Krishna R., Li H., Xiang S., Wu H., Li J., Zhou W., Chen B. (2018). Science.

[cit2] McDaniel M. P., Martin S. J. (1991). J. Phys. Chem..

[cit3] Steiner H. , Introduction to Petroleum Chemicals, Elsevier, New York, 2015, pp. 142–157

[cit4] (b) Kerry F. G. , Industrial Gas Handbook: Gas Separation and Purification, CRC Press, Boca Raton, FL, USA, 2007, pp. 134–169

[cit5] Ren T., Patel M., Blok K. (2006). Energy.

[cit6] Chen K., Madden D., Mukherjee S., Pham T., Forrest K., Kumar A., Space B., Kong J., Zhang Q., Zaworotko M. (2019). Science.

[cit7] Bloch E. D., Queen W. L., Krishna R., Zadrozny J. M., Brown C. M., Long J. R. (2012). Science.

[cit8] Suo X., Cui X., Yang L., Xu N., Huang Y., He Y., Dai S., Xing H. (2020). Adv. Mater..

[cit9] Ben T., Ren H., Ma S., Cao D., Lan J., Jing X., Wang W., Xu J., Deng F., Simmons J. M., Qiu S., Zhu G. (2009). Angew. Chem., Int. Ed..

[cit10] Tian Y., Zhu G. (2020). Chem. Rev..

[cit11] Jiang L., Tian Y., Sun T., Zhu Y., Ren H., Zou X., Ma Y., Meihaus K., Long J., Zhu G. (2018). J. Am. Chem. Soc..

[cit12] Jiang L., Wang P., Li M., Zhang P., Li J., Liu J., Ma Y., Ren H., Zhu G. (2019). Chem. Eur. J..

[cit13] Zhang P., Zou X., Song J., Tian Y., Zhu Y., Yu G., Yuan Y., Zhu G. (2020). Adv. Mater..

[cit14] Hopkinson M. N., Richter C., Schedler M., Glorius F. (2014). Nature.

[cit15] Flanigan D. M., Romanov-Michailidis F., White N. A., Rovis T. (2015). Chem. Rev..

[cit16] Zhang Y., Schmitt M., Falivene L., Caporaso L., Cavallo L., Chen E. Y.-X. (2014). J. Am. Chem. Soc..

[cit17] Magill A. M., Cavell K. J., Yates B. F. (2004). J. Am. Chem. Soc..

[cit18] Dunn M. H., Konstandaras N., Cole M. L., Harper J. B. (2017). J. Org. Chem..

[cit19] Pang J., Jiang F., Wu M., Liu C., Su K., Lu W., Yuan D., Hong M. (2015). Nat. Commun..

[cit20] Cui X., Chen K., Xing H., Yang Q., Krishna R., Bao Z., Wu H., Zhou W., Dong X., Han Y., Ren Q., Zaworotko M. J., Chen B. (2016). Science.

[cit21] Yang S., Ramirez-Cuesta A. J., Newby R., Garcia-Sakai V., Manuel P., Callear S. K., Campbell S. I., Tang C. C., Schröder M. (2015). Nat. Chem..

[cit22] Li Y. P., Wang Y., Xue Y. Y., Li H. P., Zhai Q. G., Li S. N., Jiang Y. C., Hu M. C., Bu X. H. (2019). Angew. Chem., Int. Ed..

[cit23] Xiang S., Zhou W., Gallegos J. M., Liu Y., Chen B. (2009). J. Am. Chem. Soc..

