# Supplementary material for: Tailoring the pore chemistry in porous aromatic frameworks for selective separation of acetylene from ethylene
Source: Chem Sci. 2022 Aug 20;13(37):11126–31. doi: 10.1039/d2sc03944c (PMC9516960; doi:10.1039/d2sc03944c)
Supplement: SC-013-D2SC03944C-s001 [file SC-013-D2SC03944C-s001.pdf]

Electronic Supplementary Material (ESI) for Chemical Science.
This journal is © The Royal Society of Chemistry 2022

Supporting Information

Tailoring the Pore Chemistry in Porous Aromatic Frameworks for Selective Separation of Acetylene from Ethylene

Shuang Zhou, Zhaoli Liu, Panpan Zhang, Huazhen Rong, Tingting Ma, Fengchao Cui, Dongtao Liu,* Xiaoqin Zou,* Guangshan Zhu*

Department of Chemistry, Northeast Normal University, Changchun, Jilin 130024, China

Email: liudt737@nenu.edu.cn; zouxq100@nenu.edu.cn; zhugs100@nenu.edu.cn

Table of Contents

Section S1 **Experimental Section** **Page 2**

Section S2 **Characterizations of PAF-28, cPAF-28 and iPAF-28.** **Page 11**

Section S3 **References** **Page 19**

Section S4 Cartesian Coordinates for All of the Calculated Structures **Page 21**

Section S1. Experimental Section

1 Chemicals:

All reagents and anhydrous solvents of analytical purity were commercially available and used as received unless otherwise indicated. Chemicals, 2,5-dibromotoluene (99%, Aladdin), N-bromosuccinimide (NBS, 99%, 3A Chemicals), 1-methylimidazole (99%, Acros), tetrakis (4-bromophenyl) methane (98%, jilin yanshen biochemical technology), TMS-acetylene (99%, 3A Chemicals), 1-benzyl-3-methylimidazolium chloride salt (98%, Aladdin), sodium hydride (rinse with n-hexane before used), hydrochloric acid, sodium hydroxide, dehydrated trimethylamine (99%, Aladdin), dimethylformamide (DMF), acetonitrile, methylene chloride and other solvents.

2 Synthesis of 3-(2,5-dibromobenzyl)-1-methyl-1H-imidazol-3-ium bromide:

The preparation of linear cationic building monomer (3-(2,5-dibromobenzyl)-1-methyl-1H-imidazol-3-ium bromide, DBMIIB) complied with the following route.[3]

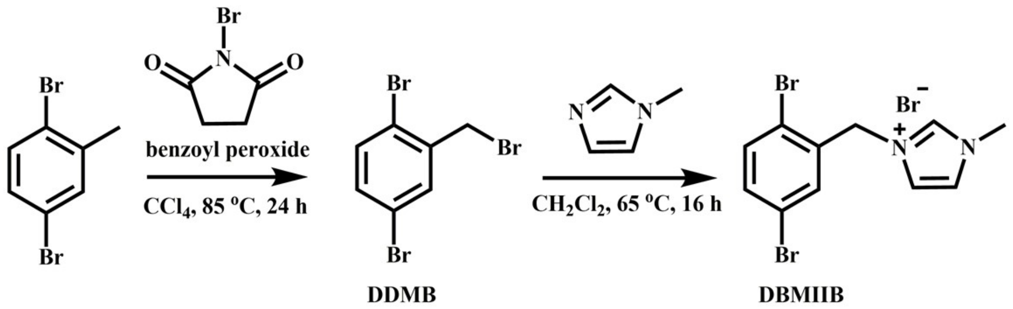

Scheme S1. Synthesis of DBMIIB.

2.1 Synthesis of 1,4-dibromo-2-(bromomethyl) benzene (DDMB):

The synthesis of DDMB followed a modified procedure from reference.[1] 1,4-Dibromo-2-methylbenzene (6.25 g, 25 mmol) was dissolved into 150 mL anhydrous CCl_4 under the stirring. This mixture was degassed with a stream of nitrogen for 10 minutes. Then, N-bromosuccinimide (4.67 g, 26.25 mmol) and benzoyl peroxide (0.302 g, 1.25 mmol) were added under N_2 protection. The mixture was heated at 85 °C under N_2 for 24 h. After being cooled, the resulting precipitate was filtered to obtain the crude compound which was recrystallized twice by petroleum ether to afford the title compound as a white solid (5.0 g, 15.2 mmol, 60% yield). ^1H NMR (500 MHz,

$CDCl_3$): δ 7.59 (1H, Ar-*H*), 7.43 (1H, Ar-*H*), 7.30 (1H, Ar-*H*), 4.53 (2H, CH_2Br).

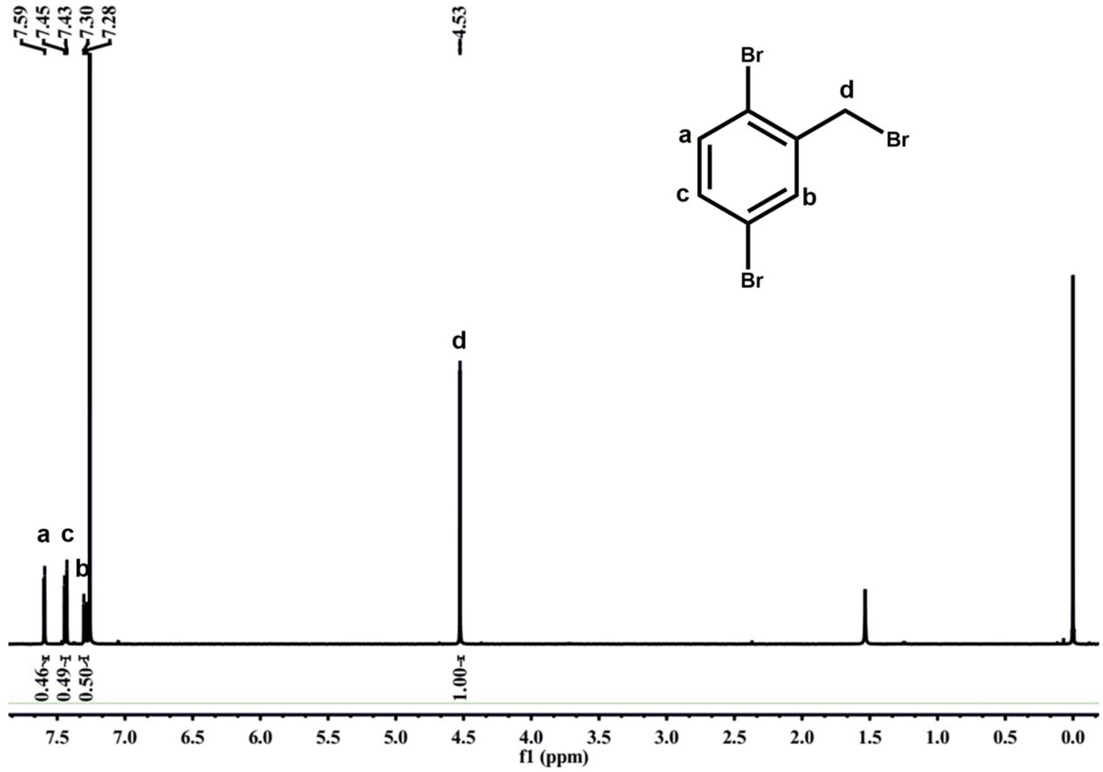

Fig. S1. ^{1}H NMR spectrum of DDMB ($CDCl_3$, 25 °C).

2.2 Synthesis of DBMIIB

The synthesis of DBMIIB followed a modified procedure from reference.[1,3] A mixture of DDMB (2.72 g, 8.3 mmol), N-methylimidazole (1.02 g, 12.4 mmol), and CH_2Cl_2 (75 mL) was heated at 65 °C under N_2 atmosphere for 24 h. After being cooled, the resulting precipitate was filtered, washed with ethyl acetate, and dried in vacuum to afford the product as a white solid (3.17 g, 7.72 mmol, 93% yield). ^{1}H NMR (500 MHz, D_2O): δ 8.7 (1H, Imidazol-*H*), 7.54 (1H, Imidazol-*H*), 7.51 (1H, Imidazol-*H*), 7.45 (1H, Ar-*H*), 7.43(1H, Ar-*H*), 7.38 (1H, Ar-*H*), 5.38 (2H, CH_2Br), 3.81 (3H, CH_3).

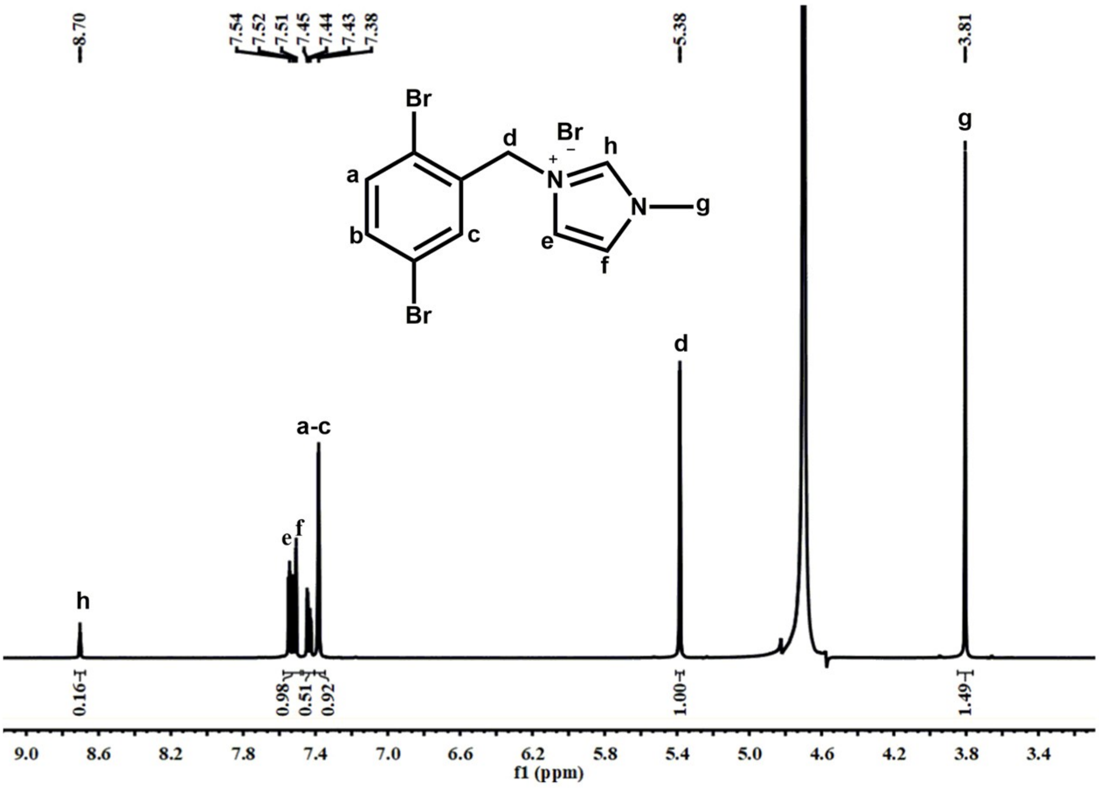

Fig. S2. ^{1}H NMR spectrum of DBMIIB (D_2O, 25 ^oC).

3 Synthesis of tetrakis(4-ethynylphenyl) methane [2]

The preparation of tetrahedral building monomer (tetrakis(4-ethynylphenyl) methane) complied with the following route.

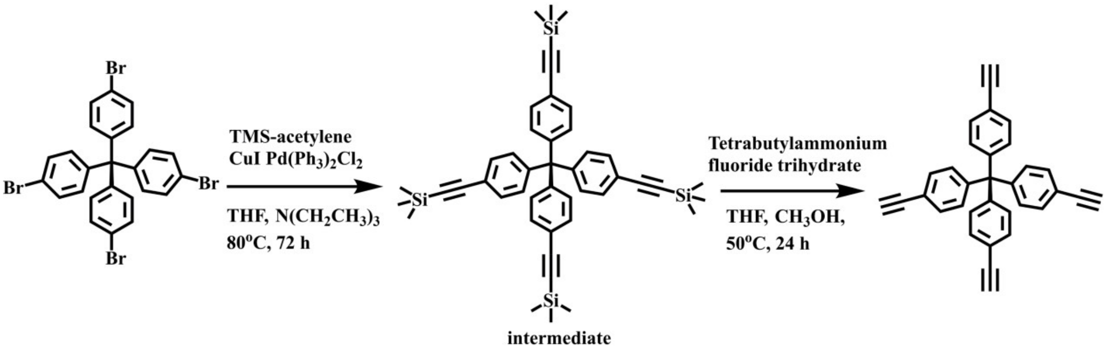

Scheme S2. Synthesis of tetrakis(4-ethynylphenyl) methane

3.1 Synthesis of intermediate

In a 100 mL round-bottomed Schlenk flask, tetrakis(4-bromophenyl) methane (1.2 g, 1.88 mmol, 1 equiv) was added to a mixture of tetrahydrofuran (5ml) and triethylamine (30ml) under a nitrogen atmosphere. Then, bis (triphenylphosphine) palladium (II) chloride (0.070 g, 0.1 mmol,

0.05 equiv), copper(I) iodide (0.025 g, 0.013 mmol, 0.07 equiv), and trimethylsilylacetylene (1.6 mL, 1g, 11.2 mmol, 6 equiv) were added to this solution subsequently. The resulting mixture was heated to 80 °C for 72 h. The organic phase was separated and washed with tetrahydrofuran. The filter liquid was concentrated under reduced pressure. After purified by column chromatography, the title compound as a yellow solid was afforded (0.82g, 70%). ^{1}H NMR (500MHz, $CDCl_3$) δ: 7.32 (8H, Ar-*H*), 7.05(8H, Ar-*H*), 0.23 (36H, SiC-H_3).

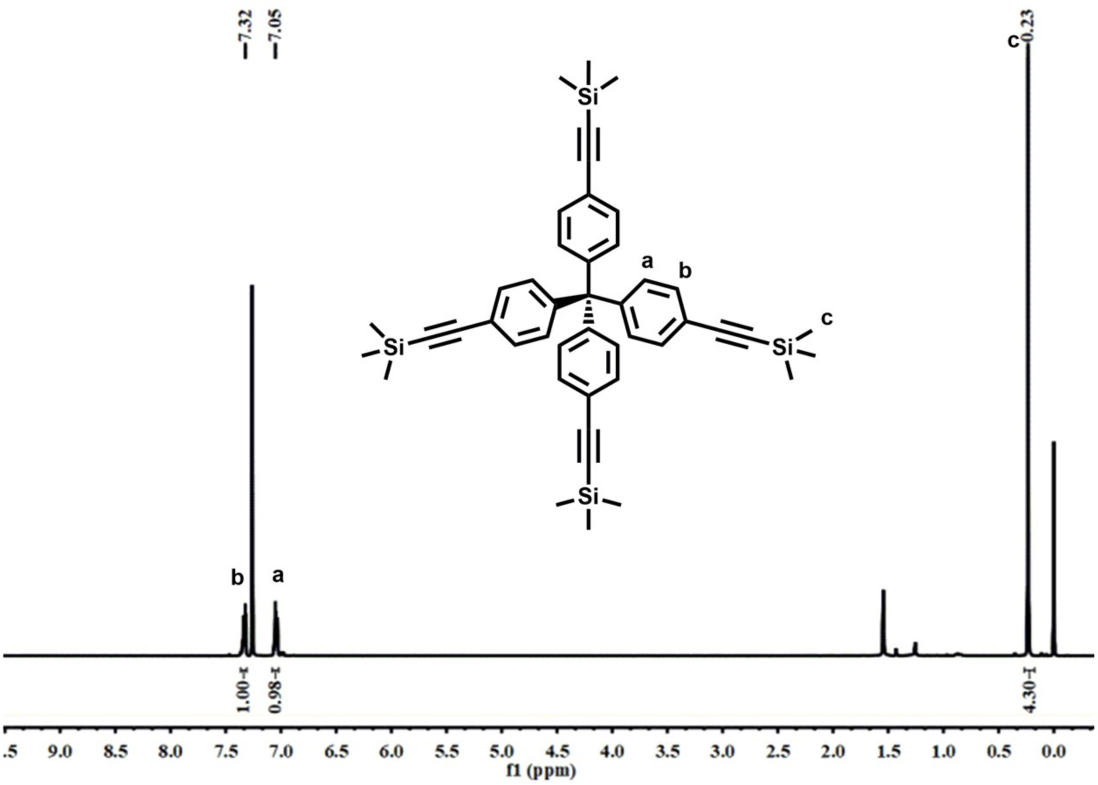

Fig. S3. ^{1}H NMR spectrum of intermediate.

3.2 Synthesis of tetrakis (4-ethynylphenyl) methane:

In a round-bottomed flask, tetrakis (4-trimethylsilylethynyl) phenylmethane (0.70 g, 1mmol,) was dissolved in a mixture of tetrahydrofuran (15 mL) and methyl alcohol (5 mL). Then tetrabutylammonium fluoride (1.85 g, 6 mmol) was added to this solution. After the reaction mixture was stirred for 24 h at 50 °C, it was poured into a saturated aqueous solution of ammonia chloride (100 mL) to remove impurities, and the pure product was obtained as a yellow solid (0.33 g, 0.80 mmol, 80%). ^{1}H NMR (500 MHz, $CDCl_3$) δ: 7.38 (8H, Ar-*H*), 7.11 (8H, Ar-*H*), 3.06 (4H, C≡C-*H*).

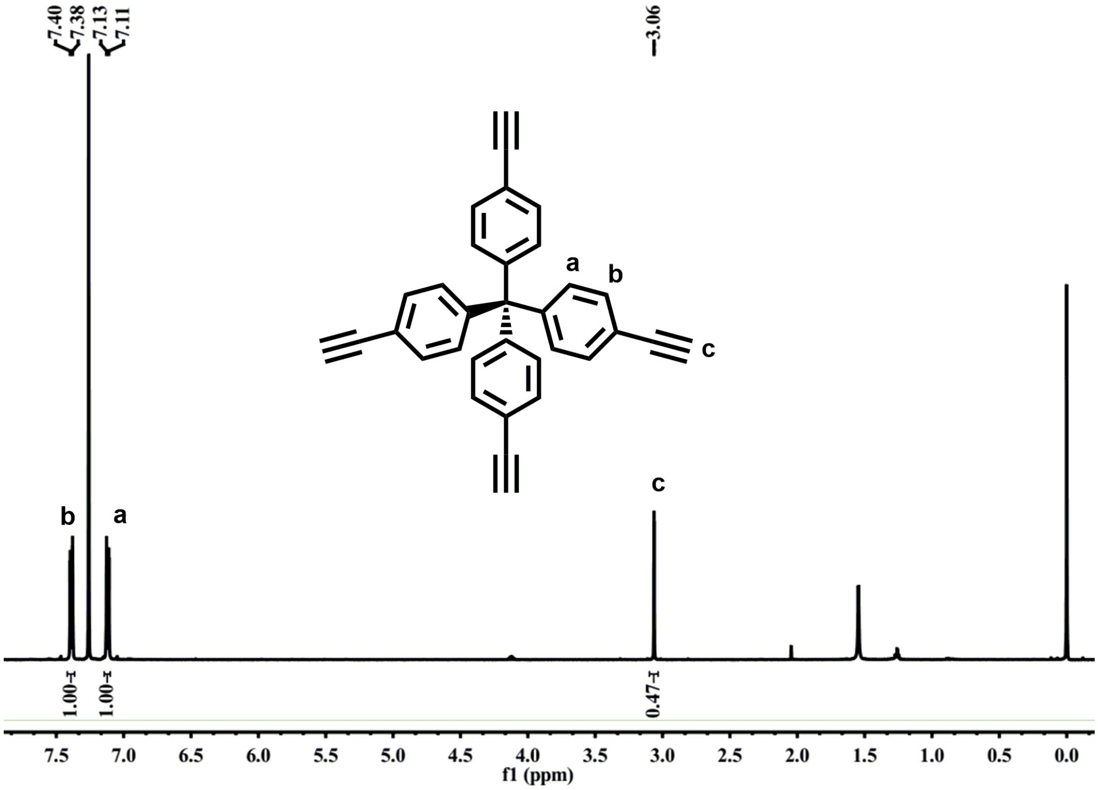

Fig. S4. ^{1}H NMR spectrum of tetrakis (4-ethynylphenyl) methane.

4 Synthesis of PAF-28:

1,4-Dibromobenzene (0.054 g, 0.23 mmol) and tetrakis (4-ethynylphenyl) methane (0.046 g, 0.11 mmol) were added to a mixture solution of bis(triphenylphosphine) palladium(II) chloride (0.070 g, 0.1 mmol), CuI (0.025 g, 0.13 mmol) in N,N-dimethylformamide (DMF) (20 mL) and triethylamine (15 mL). This mixture was degassed with a stream of nitrogen. The reaction mixture was refluxed at 100 °C for 72 h under nitrogen protection. After cooling to room temperature, the solid products were filtered and washed with chloroform, acetonitrile, DMF and methanol. Then, the products were added into 1 M HCl solution (100 mL) overnight to remove impurities from catalysts, the products were then purified by Soxhlet extraction with methanol for 48 h and subsequently dried under vacuum for 12 h at 80 °C to yield PAF-28 as a brown powder (0.054 g, 85% yields)

5 Synthesis of cPAF-28

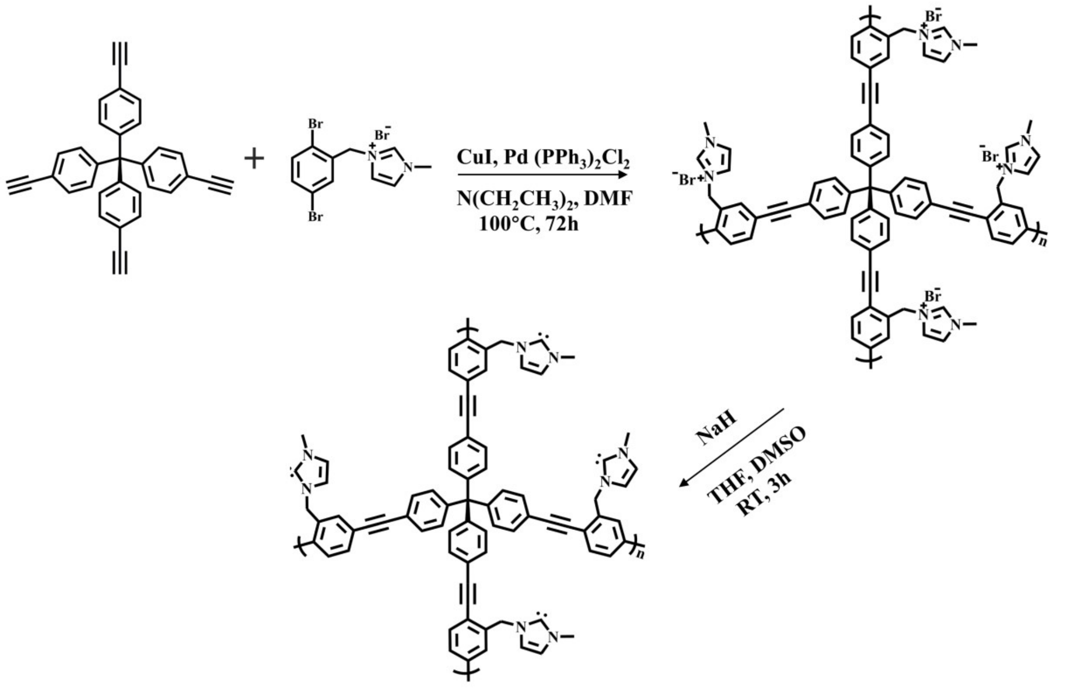

Scheme S3. The synthesis of cPAF-28.

The detail process is described below. DBMIIB (0.185 g, 0.45 mmol) and tetrakis(4-ethynylphenyl) methane (0.093 g, 0.225 mmol) were added to a mixture solution of bis(triphenylphosphine) palladium(II) chloride (0.070 g, 0.1 mmol), CuI (0.025 g, 0.13mmol) in N,N-dimethylformamide (DMF) (20 mL) and triethylamine (15 mL).[3,4] This mixture was degassed with a stream of nitrogen. The reaction mixture was refluxed at 100 °C for 72 h under nitrogen protection. After cooling to room temperature, the solid products were filtered and washed with chloroform, acetonitrile, DMF, 10% solution of hydrochloric acid and methanol. Then, the products were purified by Soxhlet extraction with methanol for 48 h and subsequently dried under vacuum for 12 h at 80 °C to yield a brown powder. The powder (0.19 g) and NaH (0.05 g) were added to a solution consist of THF and DMSO. This mixture was stirred at room temperature in the glove box for 2 hours. Then, the solid products were filtered and washed with large amounts of THF to remove NaH.[5,6] Subsequently dried under vacuum for 4 h at room temperature to yield cPAF-28 (95%, 160 mg).

6 Synthesis of iPAF-28

The materials iPAF-28 was prepared through the reaction of cPAF-28 with HCl. In detail, cPAF-28(100 mg) was added into 1 M HCl solution (100 mL) overnight and washed with water and ethanol. And then, the products were dried under vacuum for 12 h at 80 °C to yield iPAF-28 as a brown powder (94%, 110 mg).

7 General characterizations:

The morphologies of PAF products were observed with SEM (field emission scanning electron microscopy, Hitachi SU-8010) and TEM (JEM-2100F field emission electron microscopy, JEOL), and Energy-dispersive X-ray spectroscopy (EDS) images of relevant samples were obtained from scanning electron microscopy. ^{13}C NMR spectra of PAF-28, cPAF-28 and iPAF-28 were measured on a Bruker Avance III model 400 MHz solid-state nuclear magnetic resonance (NMR) spectrometer at a magic angle spinning (MAS) rate of 5 kHz. ^{1}H NMR spectra were recorded on Varian Inova 500 MHz NMR spectrometer. Infrared spectra of the monomer and the PAF product were acquired using a Nicolet IS50 Fourier transforms infrared spectrometer (FTIR). The elemental analysis (for C, H, and N) was measured using a Perkin Elmer 2400 Series II CHNS/O Analyzer. Nitrogen (N_2) and carbon dioxide (CO_2) physisorption experiments were carried out at 77 K and 298 K, and adsorption-desorption isotherms were collected on an Autosorb iQ2 adsorptometer (Quantachrome Instrument). Thermogravimetric analysis (TGA) data were obtained on a Mettler-Toledo thermal analyzer at a heating rate of 10 °C min^{-1} under air atmosphere. In-situ-IR date was measured by METTLER-TOLEDO ResctIR-15.

8 Gas adsorption of C_2H_2, C_2H_4:

Static adsorptions of C_2H_2, C_2H_4 gases over PAF-28, cPAF-28 and iPAF-28 were performed on the same instrument, an Autosorb iQ2 adsorptometer. Prior to the tests, all the samples were degassed at 120 °C for ~8 h under dynamic vacuum. High-purity gases (99.99% C_2H_2, 99.99% C_2H_4) were used for the measurements. The isotherms for each gas were collected at both 273 K and 298 K. The selectivity for one gas over another at equilibrium state was predicted using an ideal adsorbed solution theory (IAST) model, which has been widely applied for a variety of microporous materials including porous organic frameworks. The excess adsorption data for a

single-component gas of C_2H_2, C_2H_4 were first converted to absolute loadings according to Peng-Robinson equation. And then, the absolute component loadings were fitted using the dual-site Langmuir-Freundlich adsorption model (Equation S1):

$$q = q_{m1} \times \frac{b_1 \times p^{1/n_1}}{1 + b_1 \times p^{1/n_1}} + q_{m2} \times \frac{b_2 \times p^{1/n_2}}{1 + b_2 \times p^{1/n_2}} \quad \text{(S1)}$$

where p is the bulk gas pressure at equilibrium with the adsorbed phase (kPa), q is the adsorbed amount per mass of adsorbent (mmol g^{-1}), q_{m1} and q_{m2} are the saturation capacities of sites 1 and 2 (mmol g^{-1}), b_1 and b_2 are the affinity coefficients of sites 1 and 2 (kPa^{-1}), n_1 and n_2 are the deviations from an ideal homogeneous surface.

The IAST calculations were carried out for binary mixtures containing component 1 and component 2, and herein the binary gas pairs are C_2H_2/C_2H_4. The IAST selectivity can be defined in the following (Equation S2).

$$S = \frac{q_1/q_2}{p_1/p_2} \quad \text{(S2)}$$

where q_1 (q_2) and p_1 (p_2) are the mole fractions of component 1 and 2 in the adsorbed and bulk phases, respectively.[7,8,9]

9 Breakthrough experiments:

The breakthrough curves were recorded on a homemade apparatus for gas mixtures at 298 K and 1 bar. In the C_2H_2/C_2H_4 (v/v: 50/50) separation experiment, activated materials in particle form were prepared and packed into a stainless-steel column. The gas flows were controlled at the inlet by using a mass flow meter at 3 cm^3 min^{-1} and 298 K, a gas chromatograph (flame ionization detector (FID)) was used to continuously monitor the effluent gas from the adsorption bed. After each breakthrough cycling experiment, the sample in the column was regenerated with N_2 flow of 50 cm^3 min^{-1} at 353 K for 8 h.

10 Binding energy calculation:

All DFT calculations were performed by the Gaussian 16 program package. The geometries of

gases and PAF products and complexes of both were fully optimized at the B3LYP-D3/6-311++G (2d,2p) level. The binding energy (BE) was obtained by the following equation:

$$BE = E(gas + PAF\ product) - E(PAF\ product) - E(gas)$$

Where E(PAF product), E(gas) and E(gas + PAF product) are BSSE-corrected energies of the optimized PAF products, gases, and corresponding complexes of PAF products and gas; the units are kJ mol^{-1}. The negative value of BE indicates an exothermic adsorption and the higher absolute value means a higher adsorption strength.[10,11,12]

Section S2. Characterizations of PAF-28, iPAF-28-Cl and cPAF-28

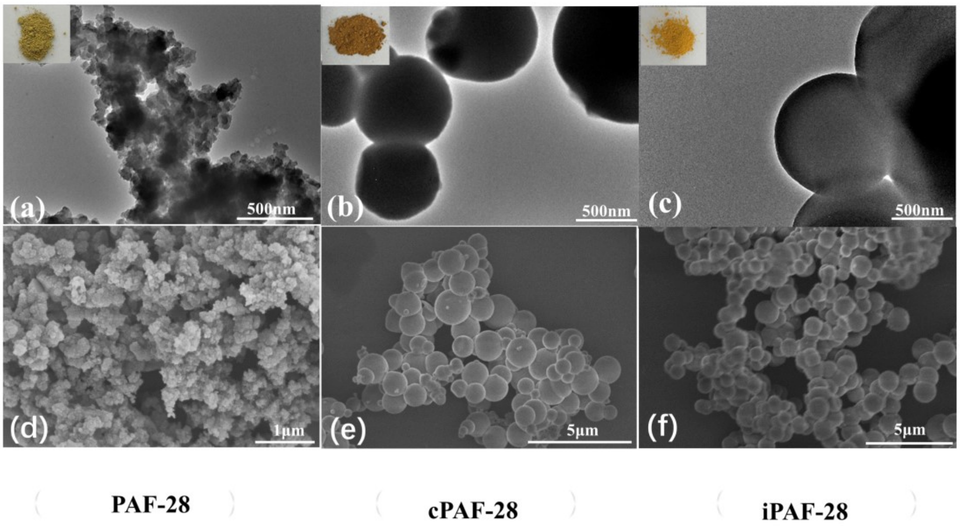

Fig. S5 TEM and SEM images of PAF-28 nanoparticles (a, d); cPAF-28 nanoparticles (b, e); iPAF-28 nanoparticles (c, f).

Table S1 Elemental analysis results (C, H, N) for PAF-28, iPAF-28 and cPAF-28.

Sample		C wt%	H wt%	N wt%
PAF-28	Calcd for $C_{45}H_{24}$	95.75	4.25	-
PAF-28	Found	87.57	4.03	-
cPAF-28	Calcd for $C_{55}H_{36}N_4$	87.76	4.79	7.44
cPAF-28	Found	79.83	4.81	6.30
iPAF-28	Calcd for $C_{55}H_{38}N_4Cl_2$	79.81	4.59	6.78
iPAF-28	Found	71.33	4.13	6.58

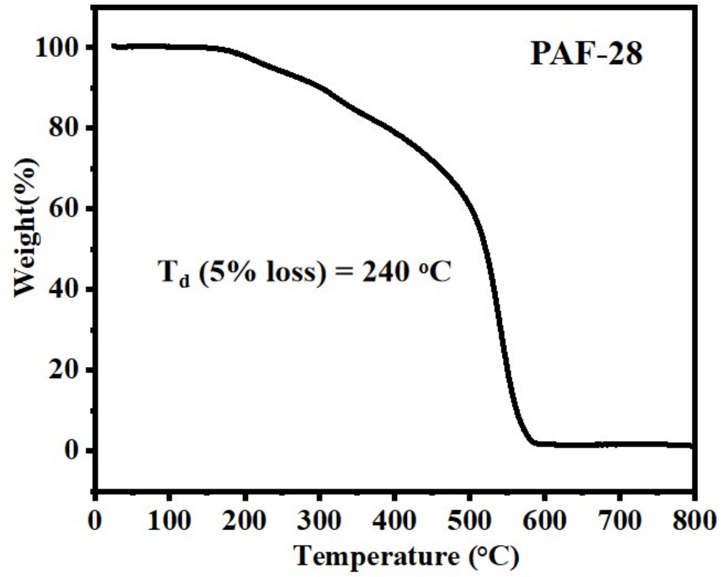

Fig. S6 TGA curve of PAF-28 under air.

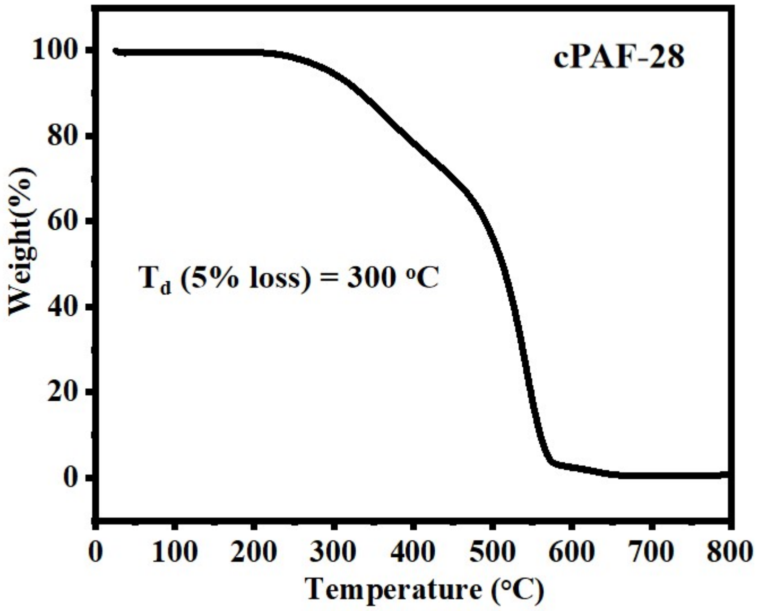

Fig. S7 TGA curve of cPAF-28 under air.

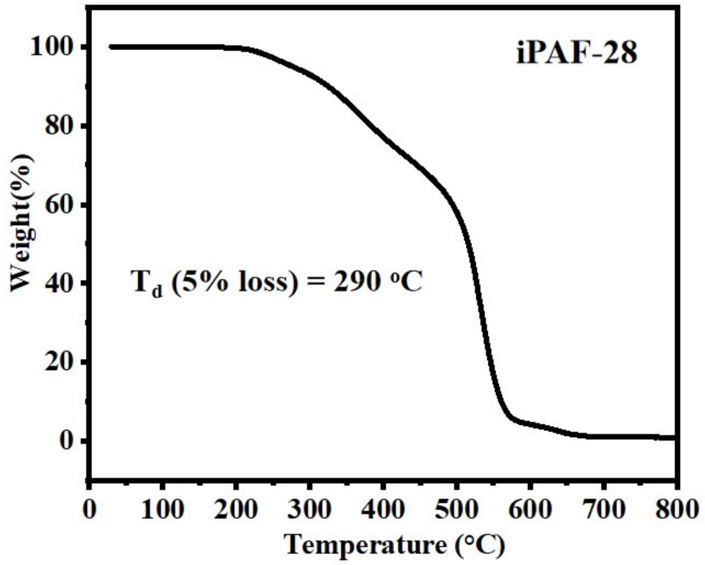

Fig. S8. TGA curve of iPAF-28 under air.

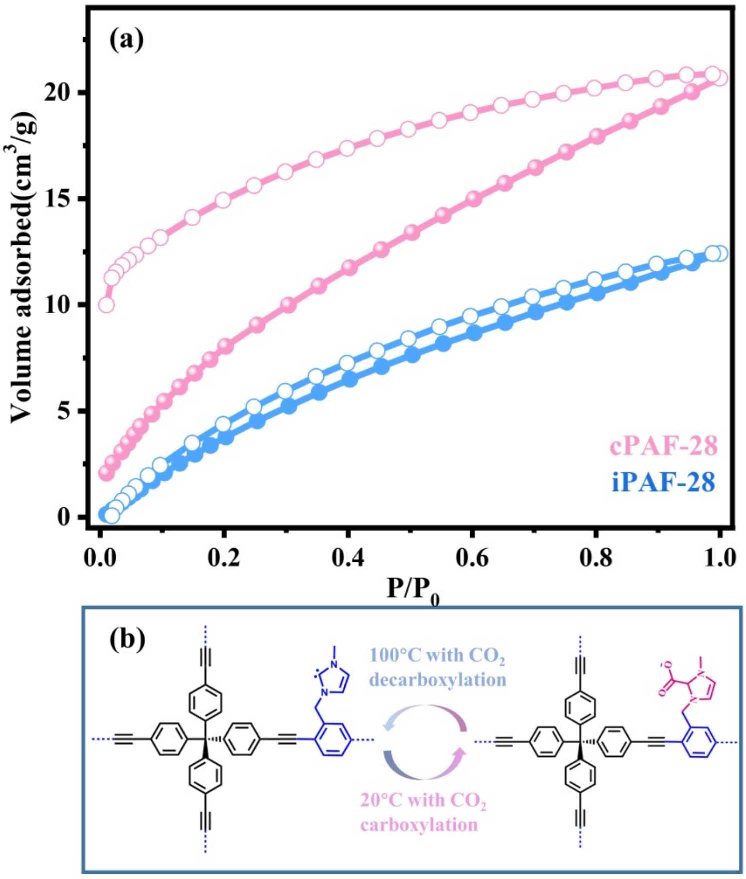

Fig. S9 CO_2 adsorption-desorption isotherms measured at 298 K for cPAF-28 and iPAF-28 materials (a) (P_0=100 kPa) and the diagram of carbene reacts with carbon dioxide (b) [13].

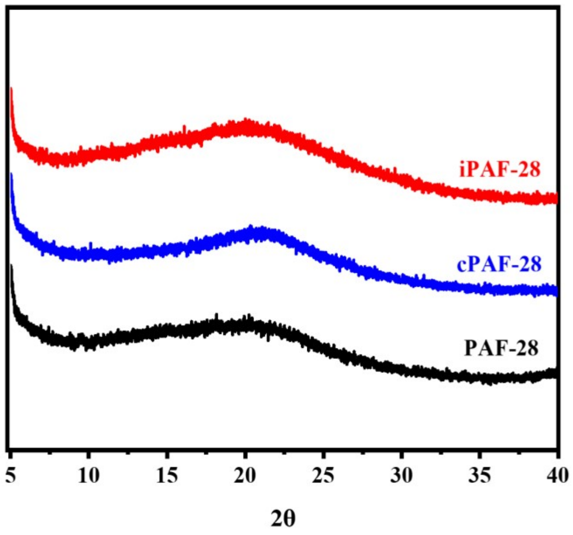

Fig. S10 PXRD spectra of PAF-28, cPAF-28 and iPAF-28 materials.

Table S2 Summary of C_2H_2 and C_2H_4 adsorption data, selectivity and heat of adsorption data for several metal-free porous organic materials (white) and some metal-organic frameworks (gray).

Sample	C_2H_2 uptake (cm^3 g^{-1})	C_2H_4 uptake (cm^3 g^{-1})	Selectivity for C_2H_2/C_2H_4 (50/50)	Q_{st} C_2H_2 (kJ mol^{-1})	Reference
**iPAF-28 (298 K)**	**48.0**	**16.8**	**15.4**	**40.0**	**this work**
PAF-110 (298 K)	50.0	28.9	5.0	38.4	[14]
PAF-120 (298 K)	50.8	27.3	4.1	37.5	[15]
iPAF-1-OH (298 K)	103.1	31.3	9.9	51.0	[16]
CTF-PO71 (298 K)	74.0	47.5	2.8	28.0	[17]
P(Ph-3MVIm-SiF_6) (298 K)	29.5	3.09	336.4	21.5	[18]
APOP (298 K)	35.8	26.2	1.8	25.8	[19]
HOF-1a (296 K)	~56	~4.0	14.6	58.1	[20]
UTSA-100a (296 K)	95.6	37.2	10.7	22	[21]
SIFSIX-2-Cu-i (298 K)	89.6	49.3	44.8 [a]	52.9	[22]
NUC-100a (298 K)	102.3	7.2	7291 [a]	60.5	[23, 24]

[a] IAST selectivities for C_2H_2/C_2H_4 (1/99) mixtures

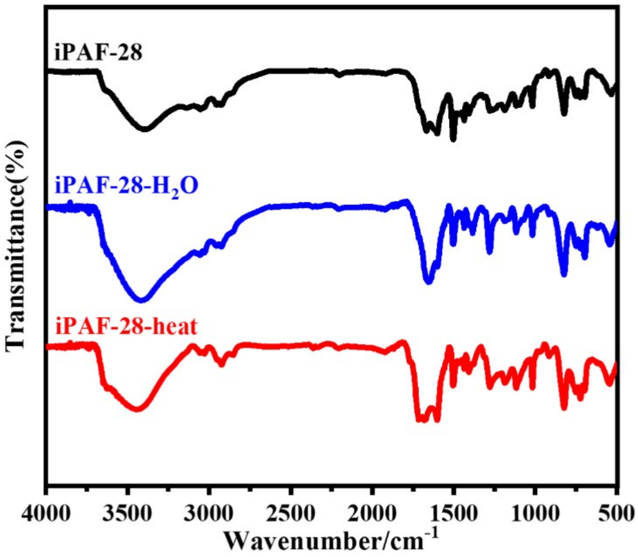

Fig. S11 FTIR spectra of iPAF-28-heat (red) that suffer from a high-temperature calcination of 200°C for 12h, iPAF-28-H_2O (blue) that immersed in water for 24h and iPAF-28 (black) that without any post-processing.

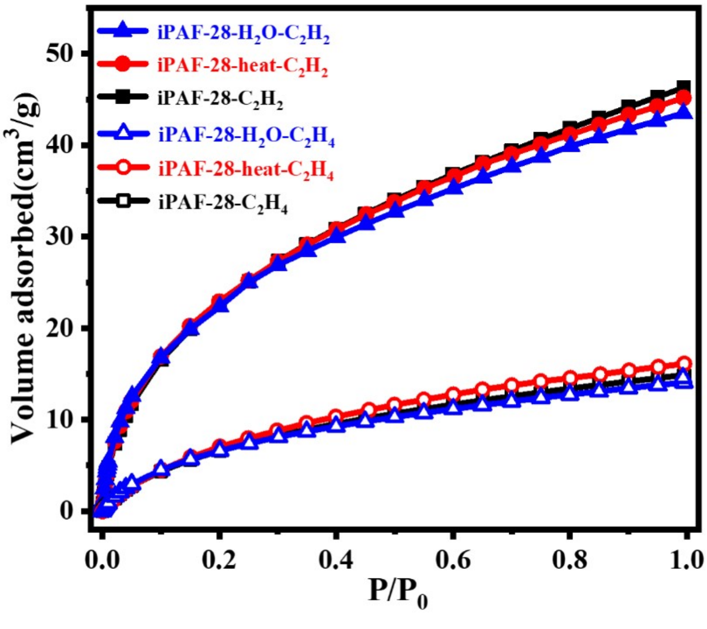

Fig. S12 C_2H_2/C_2H_4 adsorption isotherms at 298 K for iPAF-28-heat (red) that suffered from 200°C for 12h, iPAF-28-H_2O (blue) that was immersed in water for 24h and iPAF-28 (black) without any post-processing.

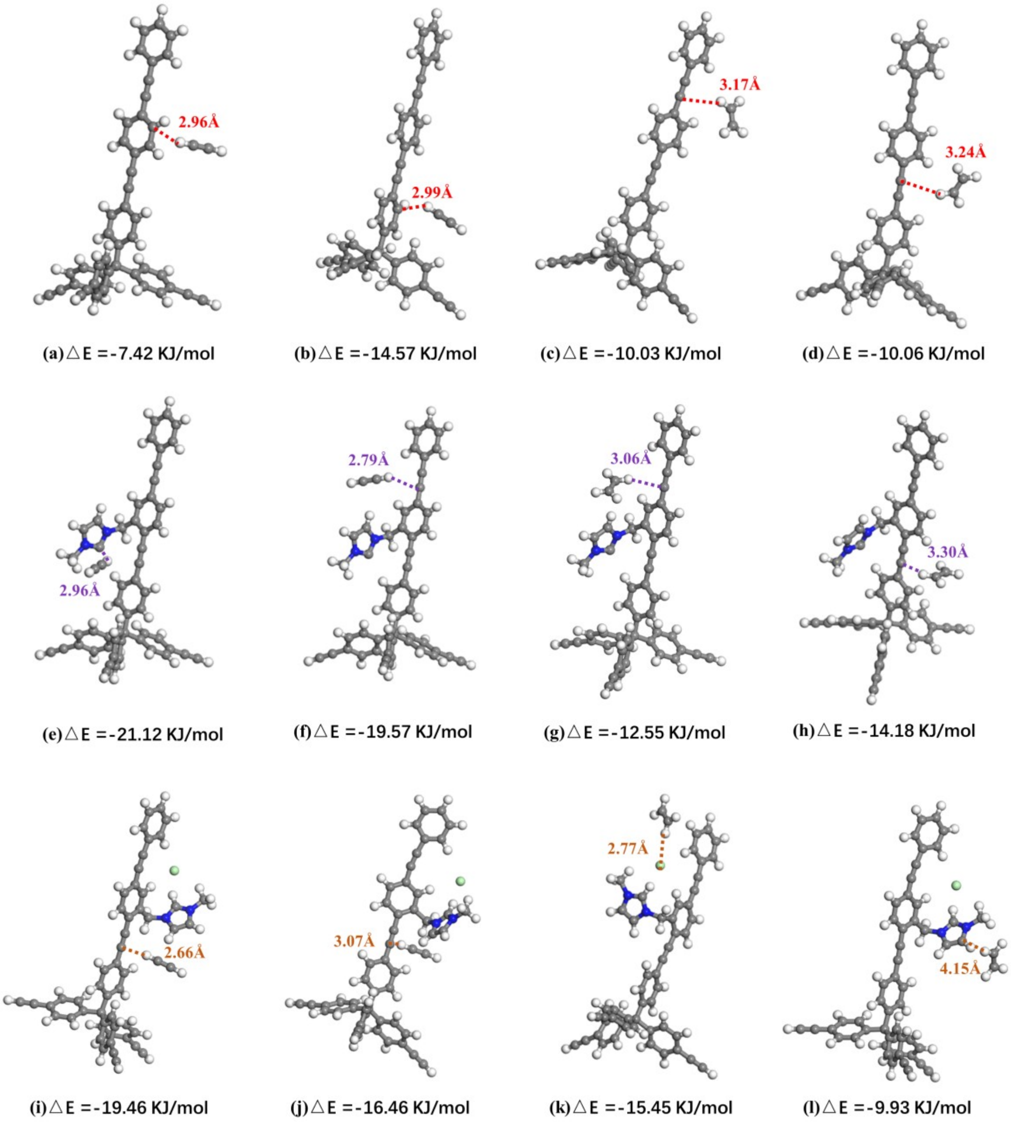

Fig. S13 Different binding sites and calculated binding energies of PAF-28 (a-d), cPAF-28 (e-h) and iPAF-28 (i-l) for C_2H_2 and C_2H_4.

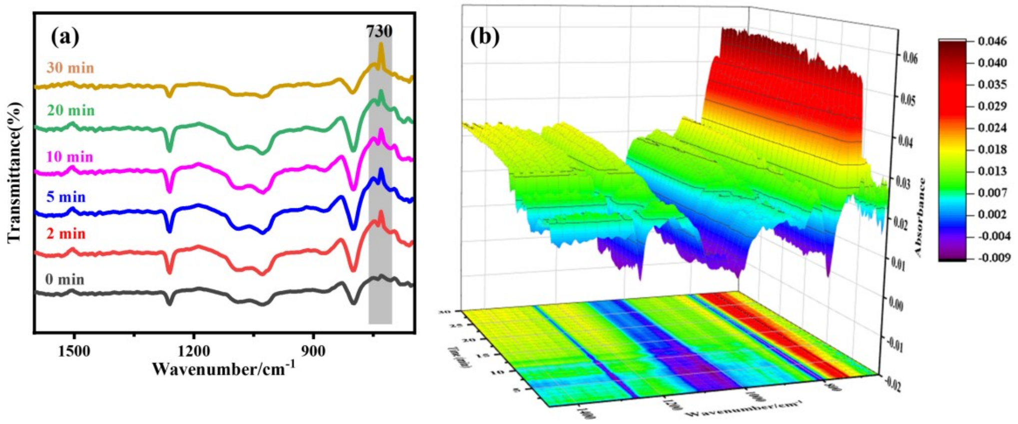

Fig. S14 In situ IR spectra of absorbance versus time for iPAF-28 - C_2H_2 (a) and 3D in situ IR spectrum (b). This varied structure of the iPAF-28 in the presence of C_2H_2 was further demonstrated by monitoring the changes of the relative intensities for the ≡C-H (730 cm^{-1}) bonds.

Section S3. References

[1] Y. Tian, J. Song, Y. Zhu, H. Zhao, F. Muhammad, T. Ma, M. Chen and G. Zhu, *Chem. Sci.* **2019**, *10*, 606-613.

[2] W. Lu, D. Yuan, D. Zhao, C. I. Schilling, O. Plietzsch, T. Muller, S. Brase, J. Guenther, J. Blumel, R. Krishna, Z. Li, and H. Zhou, *Chem. Mater.* **2010**, *22*, 5964-5972.

[3] R. Zhao, T. Ma, F. C. Cui, Y. Tian and G. Zhu, *Adv. Sci.* **2020***,* 2001899.

[4] J. Song, Y. Li, P. Cao, X. Jing, M. Faheem, Y. Matsuo, Y. Zhu, Y. Tian, X. Wang, and G. Zhu, *Adv. Mater.* **2019**, *31,* 1902444.

[5] M. L. Cole, C. Jones and P. C. Junk, *New J. Chem.* **200**2, *26*, 1296-1303.

[6] E. D-Ayoub and R. H. Grubbs, *J. Am. Chem. Soc.* **2004**, *126*, 10198-10199.

[7] R. M. Abdelhameed, H. Abdel-Gawad, C. M. Silva, J. Rocha, B. Hegazi1 and A. M. S. Silva, *Int. J. Environ. Sci. Technol.* **2018**, *15*, 2283-2294.

[8] P. Rekha, R. Muhammad, V. Sharma, M. Ramtekeb and P. Mohanty, *J. Mater. Chem.* A **2016**, *4*, 17866-17874.

[9] A. L. Myers and J. M. Prausnitz, *AIChE J.* **1965**, *11*, 121-127.

[10] A. D. Becke*, Phys. Rev. A* **1988**, *38*, 3098-3100.

[11] C. Lee, W. Yang and R. G. Parr, *Phys. Rev. B* **1988**, *37*, 785-789.

[12] E. A. Turner, C. C. Pye and R. D. Singer, *J. Phys. Chem. A* **2003**, **107**, 2277-2288.

[13] H. Zhou, W. Z. Zhang, Y. Wang, J. P. Qu and X. Lu, *Macromolecules.* **2009**, *42*, 5419-5421.

[14] L. Jiang, Y. Tian, T. Sun, Y. Zhu, H. Ren, X. Zou, Y. Ma, K.R. Meihaus, J.R. Long, G. Zhu, *J. Am. Chem. Soc.* **2018**, *140*, 15724-15730.

[15] L. Jiang, P. Wang, M. Li, P. Zhang, J. Li, J. Liu, Y. Ma, H. Ren, G. Zhu, *Chem. Eur. J.* **2019**, *25*, 9045-9051.

[16] P. Zhang, X. Zou, J. Song, Y. Tian, Y. Zhu, G. Yu, Y. Yuan, G. Zhu, *Adv. Mater.* **2020**, *32*, 1907449.

[17] Y. Lu, J. He, Y. Chen, H. Wang, Y. Zhao, Y. Han, Y. Ding, *Macromol. Rapid. Commun.* **2018**, *39*, 1700468.

[18] X. Suo, X. Cui, L. Yang, N. Xu, Y. Huang, Y. He, S. Dai, H. Xing, *Adv. Mater.* **2020**, *32*, 1907601.

[19] L. Jiang, P. Wang, Y. Wang, Y. Wang, X. Li, Q. Xia, H. Ren, *J. Colloid. Interf. Sci.* **202**1, *582*, 631-637.

[20] Y. He, S. Xiang, B. Chen, *J. Am. Chem. Soc*. **2011**, *133*, 14570.

[21] T. Hu, H. Wang, B. Li, R. Krishna, H. Wu, W. Zhou, Y. Zhao, Y. Han, X. Wang, W. Zhu, Z. Yao, S. Xiang, B. Chen, *Nat. Commun*. **2015**, *6*, 7328.

[22] X. L. Cui, K. J. Chen, H. B. Xing, Q. W. Yang, R. Krishna, Z. B. Bao, H. Wu, W. Zhou, X. L. Dong, Y. Han, B. Li, Q. L. Ren, M. J. Zaworotko, B. L. Chen, *Science* **2016**, *353*, 141−144.

[23] J. Wang, Y. Zhang, P. Zhang, J. Hu, R. Lin, Q. Deng, Z. Zeng, H. Xing, S. Deng, B. Chen, *J. Am. Chem. Soc*. **2020**, *142*, 9744-9751.

[24] R. Lin, S. Xiang, W. Zhou, B. Chen, *Chem*, **2020**, *6*, 337–363.

Section 4 Cartesian Coordinates for All of the Calculated Structures

Coordinates of Optimized Structures of PAF-28 + C_2H_2

Charge = 0 Multiplicity = 1

C 3.98908 0.05217 -0.01391	C 4.57852 -0.30946 1.3801
C 2.43616 -0.04064 -0.02371	C 4.31114 1.52222 -0.40987
C 4.63115 -0.9633 -1.00278	C 3.79945 -0.64454 2.49377
C 1.69926 0.87693 0.75037	C 5.06611 2.39632 0.38103
C 4.51128 -2.33941 -0.7293	C 5.97778 -0.36421 1.52805
C 1.71285 -0.94798 -0.80746	C 3.7675 2.03432 -1.60363
C 5.37751 -0.59269 -2.12756	C 4.38503 -0.99624 3.71014
C 0.31234 0.86881 0.76863	C 5.29168 3.71672 -0.00861
C 5.07948 -3.29977 -1.55442	C 6.57132 -0.70366 2.73573
C 0.31862 -0.95969 -0.80511	C 3.99311 3.34322 -2.0059
C 5.96087 -1.54899 -2.95903	C 5.77992 -1.02544 3.85559
C -0.41028 -0.05725 -0.01253	C 4.76605 4.21207 -1.21114
C 5.81711 -2.91861 -2.69222	C 6.38295 -1.38108 5.10119
C -1.83322 -0.06857 -0.00475	C 4.99873 5.56268 -1.61531
C 6.40992 -3.90079 -3.54401	C 6.89529 -1.68098 6.15585
C -3.05033 -0.07709 0.00332	C 5.19575 6.70605 -1.95973
C 6.91043 -4.73406 -4.26505	H 2.71918 -0.64416 2.42353
H 2.22631 1.61786 1.34297	H 5.48309 2.05876 1.32129
H 3.96873 -2.66183 0.15359	H 6.61269 -0.14473 0.67568
H 2.23213 -1.65593 -1.44083	H 3.14775 1.39498 -2.22407
H 5.52162 0.45339 -2.3661	H 3.75681 -1.2547 4.55624
H -0.22813 1.58491 1.37888	H 5.87804 4.37338 0.62568
H 4.96666 -4.35351 -1.32176	H 7.6525 -0.73281 2.82208
H -0.21526 -1.67208 -1.42551	H 3.56567 3.70928 -2.93354
H 6.53709 -1.23326 -3.82252	C -4.47209 -0.0879 0.01107
C -5.19086 -0.99948 -0.79089	C -5.19615 0.81273 0.82056

C	-6.57723	-1.00972	-0.78384	C	-6.58252	0.80222	0.82776
H	-4.65377	1.5182	1.4412	C	-7.30146	-0.1091	0.02561
H	-7.11966	-1.71492	-1.4047	H	-7.12903	1.49938	1.45411
C	-8.72325	-0.1196	0.03264	C	-9.94027	-0.12852	0.03839
C	-11.36447	-0.13888	0.04478	C	-12.08433	0.75474	0.86362
C	-12.07854	-1.0429	-0.76768	C	-13.47588	0.74074	0.86634
H	-11.53901	1.45241	1.49069	C	-13.47012	-1.0491	-0.75802
H	-11.52876	-1.73259	-1.39965	C	-14.17374	-0.15927	0.05728
H	-14.01849	1.43441	1.50182	H	-14.00824	-1.75059	-1.38872
H	-15.25956	-0.16714	0.0621	H	7.35176	-5.46705	-4.89976
H	5.36876	7.71285	-2.26192	H	7.34542	-1.94598	7.08433
H	-4.64436	-1.69659	-1.41734	C	-4.68995	-3.50589	-4.99757
C	-4.68995	-3.50589	-6.20292	H	-4.68995	-3.50589	-3.93186
H	-4.68995	-3.50589	-7.26862				

Coordinates of Optimized Structures of PAF-28 + C_2H_4

Charge = 0 Multiplicity = 1

C	-4.08963	0.12208	-0.09291	C	-4.63847	-0.3042	1.29989
C	-2.53998	0.26213	-0.07887	C	-4.39883	-0.94728	-1.18043
C	-4.78269	1.47861	-0.41129	C	-3.82684	-0.58768	2.40485
C	-1.75703	-0.89819	0.07911	C	-5.13496	-2.11383	-0.94198
C	-4.69854	2.51618	0.53701	C	-6.03281	-0.36035	1.48913
C	-1.86428	1.47407	-0.26702	C	-3.86781	-0.76925	-2.47255
C	-5.53527	1.72144	-1.56645	C	-4.37621	-0.93495	3.6394
C	-0.37057	-0.85044	0.08249	C	-5.35425	-3.05277	-1.95017
C	-5.30736	3.74586	0.33005	C	-6.59044	-0.71358	2.71018
C	-0.47129	1.53502	-0.27553	C	-4.08721	-1.69196	-3.48572
C	-6.15931	2.95077	-1.78115	C	-5.76623	-1.01107	3.81302
C	0.30253	0.37705	-0.09386	C	-4.84104	-2.85629	-3.24094
C	-6.05152	3.98671	-0.84124	C	-6.33209	-1.37041	5.0751

C 1.72441	0.42953	-0.09868		C -5.0671	-3.81403	-4.27694
C -6.68625	5.24824	-1.05916		C -6.81267	-1.67577	6.14323
C 2.94098	0.4543	-0.09579		C -5.25904	-4.62368	-5.15579
C -7.22217	6.31789	-1.24214		H -2.74916	-0.53194	2.31799
H -2.24772	-1.85947	0.19422		H -5.54158	-2.31086	0.04182
H -4.1512	2.35139	1.45966		H -6.69283	-0.11319	0.66376
H -2.42026	2.39027	-0.41997		H -3.26285	0.10664	-2.68373
H -5.65279	0.94728	-2.31392		H -3.72332	-1.14602	4.48007
H 0.20849	-1.75862	0.21658		H -5.92583	-3.9499	-1.73618
H -5.22136	4.52865	1.0765		H -7.66866	-0.75344	2.82551
H 0.02564	2.48759	-0.42767		H -3.66966	-1.52595	-4.47342
H -6.73969	3.11042	-2.68401		C 4.36219	0.45397	-0.08307
C 5.09411	1.65829	-0.14305		C 5.07195	-0.76276	-0.00837
C 6.48069	1.64529	-0.12611		C 6.45787	-0.7762	0.0096
H 4.51561	-1.68549	0.03325		C 7.19076	0.4283	-0.04878
H 7.03443	2.57714	-0.17185		H 6.99346	-1.71791	0.06789
C 8.61244	0.41544	-0.03029		C 9.82937	0.40401	-0.01436
C 11.25346	0.3902	0.00414		C 11.95744	-0.82781	0.09396
C 11.98337	1.59417	-0.0671		C 13.34893	-0.83623	0.1117
H 11.39992	-1.7571	0.14898		C 13.37477	1.57534	-0.04867
H 11.44589	2.53416	-0.13636		C 14.06254	0.3627	0.04062
H 13.87917	-1.78143	0.18121		H 13.92517	2.50995	-0.10416
H 15.14827	0.35207	0.05472		H -7.69418	7.25948	-1.40216
H -5.42942	-5.33692	-5.92855		H -7.2327	-1.943	7.0852
H 4.55876	2.60005	-0.20141		C 1.89915	-4.47156	0.8885
C 2.11742	-3.55219	1.8262		H 2.66514	-4.75462	0.17166
H 0.94597	-4.98591	0.80228		H 1.35155	-3.27463	2.54509
H 3.07084	-3.03952	1.91838				

Coordinates of Optimized Structures of cPAF-28 + C_2H_2

Charge = 0   Multiplicity = 3

C	4.32114	-1.19075	0.18506	C	4.62567	-1.87115	1.55176
C	2.92499	-0.50266	0.19217	C	5.3473	-0.06323	-0.12862
C	4.38871	-2.32854	-0.87521	C	3.7921	-1.7765	2.67236
C	2.74474	0.64862	0.98401	C	6.46897	0.21459	0.66157
C	3.64384	-3.50331	-0.65549	C	5.78042	-2.66864	1.66879
C	1.83615	-0.93753	-0.57408	C	5.12483	0.7609	-1.24879
C	5.18233	-2.27584	-2.02719	C	4.10355	-2.42867	3.86557
C	1.53034	1.31811	1.03043	C	7.34379	1.25308	0.3419
C	3.66696	-4.56117	-1.55358	C	6.10482	-3.31495	2.85349
C	0.61047	-0.27286	-0.54514	C	5.99326	1.7912	-1.5816
C	5.21999	-3.33641	-2.93253	C	5.26723	-3.20369	3.98031
C	0.43879	0.8648	0.26342	C	7.12607	2.05578	-0.78771
C	4.45954	-4.49502	-2.71589	C	5.59034	-3.87052	5.20216
C	-0.81602	1.53764	0.2777	C	8.02177	3.11853	-1.11958
C	4.49426	-5.58238	-3.64235	C	5.86595	-4.43512	6.23664
C	-1.91019	2.06981	0.24026	C	8.78004	4.01842	-1.40254
C	4.52208	-6.50381	-4.42659	H	2.87827	-1.19824	2.62502
H	3.57631	1.02989	1.56799	H	6.67132	-0.37316	1.54786
H	3.04019	-3.59146	0.242	H	6.43212	-2.79132	0.80972
H	1.93319	-1.80389	-1.216	H	4.24779	0.59547	-1.8662
H	5.79216	-1.40478	-2.23014	H	3.43682	-2.34113	4.71713
H	1.41826	2.20088	1.65164	H	8.20307	1.44836	0.97508
H	3.07921	-5.45251	-1.36024	H	7.00372	-3.91946	2.91586
H	-0.23476	-0.6302	-1.12998	H	5.79829	2.40731	-2.4532
H	5.84748	-3.26965	-3.81524	C	-3.19291	2.67051	0.12733
C	-4.07315	2.30172	-0.92501	C	-3.60048	3.66121	1.04327
C	-5.30579	2.93767	-1.03021	C	-3.68119	1.24088	-1.9348
C	-4.83758	4.27667	0.93172	H	-2.92412	3.94149	1.84384
C	-5.71611	3.92734	-0.1131	H	-5.97374	2.67677	-1.84619
H	-4.26073	1.38544	-2.85349	H	-2.62187	1.3121	-2.18415

N	-3.88073	-0.13133	-1.46086	H	-5.13605	5.03755	1.64493
C	-5.1135	-0.65649	-1.08431	C	-2.86988	-1.05866	-1.42223
C	-4.89016	-1.96027	-0.78703	H	-6.01958	-0.07205	-1.04968
N	-3.53303	-2.17946	-0.99946	H	-5.5655	-2.72989	-0.44559
C	-2.86783	-3.45131	-0.76186	H	-2.8291	-3.67477	0.30872
H	-3.38836	-4.25991	-1.2842	H	-1.85182	-3.36907	-1.1464
C	-6.98307	4.55843	-0.24672	C	-8.06752	5.09884	-0.36158
C	-9.33549	5.73354	-0.49569	C	-9.72197	6.7573	0.39301
C	-10.22504	5.34975	-1.5197	C	-10.96124	7.37563	0.25721
H	-9.0406	7.05629	1.18272	C	-11.4623	5.9739	-1.64702
H	-9.93197	4.56246	-2.20648	C	-11.83529	6.98766	-0.76116
H	-11.24667	8.16328	0.94824	H	-12.1383	5.66894	-2.44038
H	-12.8014	7.47254	-0.86391	H	4.54776	-7.31425	-5.11749
H	9.44749	4.81079	-1.65054	H	6.10779	-4.93207	7.14734
C	-0.69197	-0.1165	-4.02241	C	0.02466	-0.04247	-4.99031
H	-1.33568	-0.1868	-3.17618	H	0.67097	0.0291	-5.83885

Coordinates of Optimized Structures of cPAF-28 + C_2H_4

Charge = 0 Multiplicity = 3

C	-4.39919	-0.57128	0.05257	C	-5.01802	-0.79152	1.4635
C	-2.8441	-0.61471	0.0945	C	-4.81239	-1.6984	-0.9372
C	-4.92485	0.81853	-0.40932	C	-4.26319	-1.03495	2.617
C	-2.2135	-1.83655	0.40045	C	-5.66494	-2.75737	-0.60384
C	-4.71767	1.93106	0.42844	C	-6.41414	-0.68532	1.61382
C	-2.02247	0.4792	-0.20834	C	-4.25637	-1.69667	-2.23096
C	-5.65169	1.02584	-1.58759	C	-4.87069	-1.19029	3.86317
C	-0.83142	-1.95493	0.43555	C	-5.97033	-3.76093	-1.52355
C	-5.184	3.19422	0.0929	C	-7.0302	-0.84688	2.8469
C	-0.63167	0.37458	-0.18608	C	-4.56048	-2.68425	-3.15717
C	-6.13286	2.28944	-1.93089	C	-6.2642	-1.10631	4.00009

C	-0.01413	-0.84471	0.14343
C	-5.90253	3.39686	-1.10136
C	1.40577	-0.93688	0.16528
C	-6.3919	4.69294	-1.45132
C	2.62244	-0.9573	0.17161
C	-6.80585	5.79127	-1.74646
H	-2.82015	-2.71274	0.60608
H	-4.18723	1.79996	1.36605
H	-2.45853	1.43332	-0.47683
H	-5.86015	0.19746	-2.25259
H	-0.37247	-2.90801	0.6781
H	-5.00522	4.03486	0.75514
H	-0.00424	1.23321	-0.41429
H	-6.69641	2.42085	-2.84876
C	4.75018	0.14912	-0.41769
C	6.14101	0.12283	-0.42829
C	6.15843	-2.02209	0.6938
C	6.87233	-0.94969	0.12348
H	4.70698	1.87939	-1.67662
N	3.44106	2.24287	-0.04401
C	4.1341	2.7735	1.03977
C	3.27941	3.6345	1.64572
N	2.1029	3.59912	0.90359
C	0.91197	4.3688	1.2258
H	1.14979	5.43479	1.29872
C	8.29381	-0.94449	0.1014
C	10.93488	-0.93072	0.06482
C	11.63859	0.1547	-0.49542
H	11.12728	-2.84646	1.03779
H	11.08112	0.98649	-0.91341
C	-5.42978	-3.73938	-2.81777
C	-6.89018	-1.26934	5.2742
C	-5.74345	-4.76337	-3.76373
C	-7.42234	-1.40823	6.35233
C	-6.00891	-5.62912	-4.56674
H	-3.18412	-1.09865	2.5582
H	-6.09694	-2.81775	0.38694
H	-7.02821	-0.46238	0.74717
H	-3.56431	-0.90942	-2.5121
H	-4.26054	-1.37539	4.74117
H	-6.63147	-4.57217	-1.23692
H	-8.1088	-0.76487	2.93177
H	-4.12101	-2.65485	-4.14877
C	4.04239	-0.93999	0.15793
C	4.77218	-2.01432	0.70577
C	4.01699	1.32456	-1.03062
H	4.22785	-2.84611	1.14031
H	6.68648	0.94652	-0.87964
H	3.17676	0.99036	-1.6417
H	6.70421	-2.85687	1.12045
C	2.17021	2.74135	-0.15947
H	5.14438	2.49013	1.28983
H	3.40449	4.24829	2.5248
H	0.47622	4.0389	2.17478
H	0.18884	4.21678	0.42518
C	9.51076	-0.938	0.08436
C	11.66467	-2.00869	0.60574
C	13.05606	-1.99671	0.58458
C	13.03008	0.15741	-0.51166
C	13.74366	-0.91596	0.02711

H	13.60656	-2.83343	1.00438	H	13.56031	0.99962	-0.94632
H	14.82939	-0.91031	0.01247	H	-7.17033	6.7576	-2.00758
H	-6.24215	-6.39185	-5.27293	H	-7.89011	-1.53012	7.30163
C	-0.12133	3.97354	-2.81533	C	-1.17122	3.22729	-3.15385
H	0.68276	3.58385	-2.19313	H	-0.03673	5.00671	-3.14603
H	-1.98179	3.61214	-3.76773	H	-1.25994	2.19309	-2.83076

Coordinates of Optimized Structures of iPAF-28 + C_2H_2

Charge = 0 Multiplicity = 1 in supermolecule

Charge = 0 Multiplicity = 1 in fragment 1.

Charge = 0 Multiplicity = 1 in fragment 2.

C	-5.36652	0.25216	0.00174	C	-5.88246	-0.70269	-1.11411
C	-3.81517	0.37322	-0.02493	C	-5.89801	1.70197	-0.19643
C	-5.86807	-0.36139	1.34159	C	-5.60332	-2.07916	-1.00952
C	-2.98999	-0.35178	-0.89355	C	-5.43034	2.45028	-1.29404
C	-5.031	-0.67734	2.41826	C	-6.65536	-0.28438	-2.20383
C	-3.19293	1.30335	0.83112	C	-6.78544	2.33446	0.68239
C	-7.23686	-0.66439	1.47595	C	-6.04612	-2.98878	-1.96008
C	-1.60673	-0.17428	-0.89972	C	-5.84986	3.75393	-1.52114
C	-5.53423	-1.25011	3.58681	C	-7.11324	-1.19083	-3.16072
C	-1.81729	1.48214	0.84137	C	-7.20732	3.64719	0.4688
C	-7.75005	-1.22642	2.63692	C	-6.8121	-2.55735	-3.06059
C	-0.99083	0.74125	-0.02994	C	-6.75307	4.37835	-0.63889
C	-6.90246	-1.5293	3.72037	C	-7.27616	-3.48817	-4.04024
C	0.42047	0.93187	-0.02938	C	-7.18575	5.72152	-0.86282
C	-7.42168	-2.11257	4.91692	C	-7.66668	-4.27812	-4.86942
C	1.62416	1.12402	-0.01376	C	-7.55172	6.85868	-1.05514
C	-7.86301	-2.60554	5.92998	H	-5.03521	-2.44541	-0.1596
H	-3.42088	-1.05962	-1.59081	H	-4.71703	2.00319	-1.97994
H	-3.96645	-0.48782	2.35658	H	-6.92088	0.75949	-2.3165

H	-3.80352	1.90691	1.49579	H	-7.15521	1.81156	1.55567
H	-7.91197	-0.46204	0.64994	H	-5.81255	-4.04377	-1.85505
H	-0.99683	-0.74314	-1.59552	H	-5.476	4.3049	-2.37854
H	-4.86082	-1.48701	4.40477	H	-7.71423	-0.83813	-3.99314
H	-1.36724	2.20504	1.51467	H	-7.89354	4.11355	1.1689
H	-8.81067	-1.44482	2.71331	C	3.02868	1.35417	0.01541
C	3.55075	2.42889	0.76675	C	3.93374	0.52117	-0.69064
C	4.91611	2.66171	0.83227	H	2.86226	3.07488	1.30233
C	5.30166	0.75811	-0.61933	C	3.43993	-0.65358	-1.50655
C	5.81902	1.82303	0.14746	H	5.2993	3.48999	1.41987
H	6.00537	0.10547	-1.13543	H	2.38275	-0.55136	-1.75724
H	4.01116	-0.74975	-2.43224	N	3.59745	-1.93789	-0.78733
C	2.73037	-2.47528	0.15004	C	4.69736	-2.69912	-0.85235
C	3.33174	-3.59294	0.64667	H	1.78429	-2.01041	0.37966
N	4.55567	-3.71153	0.00954	H	5.63559	-2.48398	-1.39905
H	3.00039	-4.30423	1.38675	C	5.593	-4.71997	0.25659
H	5.88254	-4.68048	1.30831	H	5.21487	-5.71075	-0.0076
H	6.45936	-4.45982	-0.35662	Cl	7.70192	-2.09523	-1.53856
C	7.22613	1.99351	0.20361	C	8.44234	2.00084	0.17667
C	9.86095	1.91968	0.10152	C	10.44997	0.78296	-0.49311
C	10.68416	2.94563	0.60523	C	11.83648	0.68892	-0.57665
H	9.81065	-0.00999	-0.87471	C	12.06887	2.83812	0.51349
H	10.22692	3.81809	1.06253	C	12.64803	1.71153	-0.07707
H	12.28462	-0.18884	-1.03407	H	12.6983	3.63386	0.90314
H	13.72973	1.63098	-0.14658	H	-8.25203	-3.03977	6.82284
H	-8.01325	-4.97292	-5.60035	H	-7.87427	7.86103	-1.22351
C	6.94251	-2.40226	3.10917	C	7.30037	-2.21292	1.97067
H	6.65505	-2.54	4.12695	H	7.59684	-2.064	0.94155

Coordinates of Optimized Structures of iPAF-28 + C_2H_4

Charge = 0 Multiplicity = 1 in supermolecule

Charge = 0 Multiplicity = 1 in fragment 1.

Charge = 0 Multiplicity = 1 in fragment 2.

C	5.3703	-0.26008	-0.03304	C	5.79905	1.04599	-0.76271
C	3.82477	-0.43874	-0.04734	C	5.93517	-1.52634	-0.74097
C	5.9191	-0.12163	1.41678	C	5.46303	2.285	-0.18373
C	2.93849	0.48305	-0.61805	C	5.45055	-1.8589	-2.02072
C	5.12355	-0.20702	2.56561	C	6.54824	1.07019	-1.94512
C	3.27304	-1.62084	0.48473	C	6.86499	-2.3962	-0.15888
C	7.28728	0.15981	1.597	C	5.82815	3.48857	-0.77102
C	1.56323	0.25281	-0.6378	C	5.8946	-2.98508	-2.70002
C	5.66697	-0.04327	3.84028	C	6.92804	2.27394	-2.53989
C	1.906	-1.85652	0.4811	C	7.31196	-3.53561	-0.82871
C	7.84039	0.31607	2.86048	C	6.56966	3.50406	-1.96861
C	1.01707	-0.91635	-0.08199	C	6.84069	-3.8476	-2.11271
C	7.03512	0.21451	4.01173	C	6.95317	4.73862	-2.5771
C	-0.38655	-1.15587	-0.08874	C	7.299	-5.01173	-2.80276
C	7.59585	0.37911	5.31556	C	7.27557	5.78522	-3.09167
C	-1.58341	-1.38699	-0.08249	C	7.68673	-5.99658	-3.38908
C	8.07265	0.51781	6.41897	H	4.91113	2.30494	0.75136
H	3.31449	1.39378	-1.06791	H	4.70434	-1.22528	-2.49058
H	4.06026	-0.39543	2.48141	H	6.8552	0.14463	-2.41642
H	3.93225	-2.37552	0.90287	H	7.2489	-2.1999	0.83458
H	7.92942	0.26686	0.72787	H	5.55196	4.42885	-0.30395
H	0.9046	0.98359	-1.0982	H	5.50681	-3.21353	-3.68789
H	5.02527	-0.1125	4.71324	H	7.512	2.26239	-3.45497
H	1.51084	-2.77491	0.90402	H	8.03142	-4.19388	-0.35166
H	8.89964	0.52757	2.96873	C	-2.98031	-1.65941	-0.05877
C	-3.44928	-2.89455	0.43845	C	-3.93073	-0.70969	-0.51311
C	-4.80626	-3.17167	0.50737	H	-2.72594	-3.62932	0.77772

C	-5.28982	-0.99317	-0.44191	C	-3.49606	0.63852	-1.04613
C	-5.75369	-2.21925	0.07948	H	-5.14798	-4.12368	0.90128
H	-6.02869	-0.25903	-0.76327	H	-2.44389	0.63617	-1.33511
H	-4.09642	0.92525	-1.9122	N	-3.67489	1.71288	-0.04364
C	-2.78275	2.08074	0.95046	C	-4.82383	2.37974	0.12912
C	-3.4192	3.00241	1.72769	H	-1.7974	1.64689	1.01906
N	-4.69022	3.16649	1.20212	H	-5.78268	2.24479	-0.41662
H	-3.08323	3.54764	2.59574	C	-5.76229	4.03853	1.69762
H	-5.76321	4.01113	2.78912	H	-5.60646	5.05824	1.3386
H	-6.70739	3.65165	1.30493	Cl	-7.80804	1.93418	-0.68894
C	-7.15476	-2.42414	0.165	C	-8.37095	-2.42602	0.19908
C	-9.78986	-2.32093	0.21675	C	-10.3824	-1.07338	-0.07655
C	-10.60799	-3.42684	0.51929	C	-11.76931	-0.95125	-0.06483
H	-9.74613	-0.22009	-0.3035	C	-11.99303	-3.28879	0.52609
H	-10.14708	-4.38416	0.74429	C	-12.57649	-2.05266	0.23408
H	-12.22084	0.01083	-0.29077	H	-12.61928	-4.14602	0.75893
H	-13.65848	-1.94883	0.24007	H	8.49278	0.64	7.39141
H	7.5621	6.70644	-3.54596	H	8.0283	-6.86515	-3.90483
C	-5.54903	5.74479	-1.8739	C	-6.82053	5.55104	-1.52185
H	-5.09929	6.73516	-1.91143	H	-4.90601	4.91541	-2.16289
H	-7.27043	4.55907	-1.48774	H	-7.46159	6.38869	-1.25181
